# Tailoring Iron Oxide Nanoparticles for Efficient Cellular Internalization and Endosomal Escape

**DOI:** 10.3390/nano10091816

**Published:** 2020-09-11

**Authors:** Laura Rueda-Gensini, Javier Cifuentes, Maria Claudia Castellanos, Paola Ruiz Puentes, Julian A. Serna, Carolina Muñoz-Camargo, Juan C. Cruz

**Affiliations:** 1 Department of Biomedical Engineering, School of Engineering, Universidad de Los Andes, Carrera 1 No. 18A-12, 111711 Bogotá, Colombia; l.ruedag@uniandes.edu.co (L.R.-G.); jf.cifuentes10@uniandes.edu.co (J.C.); mc.castellanos10@uniandes.edu.co (M.C.C.); p.ruiz@uniandes.edu.co (P.R.P.); ja.serna10@uniandes.edu.co (J.A.S.); 2 School of Chemical Engineering and Advanced Materials, The University of Adelaide, Adelaide 5005, Australia

**Keywords:** iron oxide nanoparticles, endocytosis, endosomal escape, drug delivery

## Abstract

Iron oxide nanoparticles (IONs) have been widely explored for biomedical applications due to their high biocompatibility, surface-coating versatility, and superparamagnetic properties. Upon exposure to an external magnetic field, IONs can be precisely directed to a region of interest and serve as exceptional delivery vehicles and cellular markers. However, the design of nanocarriers that achieve an efficient endocytic uptake, escape lysosomal degradation, and perform precise intracellular functions is still a challenge for their application in translational medicine. This review highlights several aspects that mediate the activation of the endosomal pathways, as well as the different properties that govern endosomal escape and nuclear transfection of magnetic IONs. In particular, we review a variety of ION surface modification alternatives that have emerged for facilitating their endocytic uptake and their timely escape from endosomes, with special emphasis on how these can be manipulated for the rational design of cell-penetrating vehicles. Moreover, additional modifications for enhancing nuclear transfection are also included in the design of therapeutic vehicles that must overcome this barrier. Understanding these mechanisms opens new perspectives in the strategic development of vehicles for cell tracking, cell imaging and the targeted intracellular delivery of drugs and gene therapy sequences and vectors.

## 1. Introduction

Iron oxide nanoparticles (IONs) have gained significant attention over the past decades for their promising performance in disease diagnostics and the delivery of therapeutics [1]. In particular, their exceptional magnetic properties have alleviated some of the most relevant shortcomings in the targeted delivery of nanovehicles for biomedical applications [2]. IONs can be precisely guided and accumulated in specific tissues with the application of external magnetic fields since, at nanoscale sizes, iron oxide exhibits superparamagnetic properties that stem from its inherent ferromagnetism [3]. Iron oxide atoms exhibit strong magnetic dipoles, meaning their individual magnetic moments are prone to be coupled and create subdomain regions with a single magnetic moment, called Weiss domains [4]. Alignment of all Weiss domains within a metal structure results in strong magnetization, but this is difficult to manipulate in large multi-domain structures. However, when particle size is reduced below 100 nm, each nanoparticle behaves as a single Weiss domain and can be reversibly magnetized in a paramagnetic manner [5]. Accordingly, the alignment of IONs, upon exposure to an external magnetic field, creates a single large magnetic domain with superior net magnetization than regular paramagnetic materials, allowing their manipulation with high spatial resolution [4].

In addition to their remarkable magnetic properties, the low toxicity profile of IONs has made them superior candidates for biomedical applications when compared to other metal oxide nanoparticles [6,7,8]. Since iron is an essential element in the body, iron (III) ions released from iron oxide nanostructures have been proven to eventually incorporate back into the natural cell metabolism [9] and they have even been prescribed for treating iron deficiencies [10]. The broad versatility of modifications that can be performed on ION surfaces is highly attractive, as well, since they can be readily tuned to interact with different cell lines and subcellular compartments. Similarly, it allows the immobilization of several targeting agents and therapeutic molecules that can dictate numerous functionalities [11]. This makes them suitable for a wide variety of biomedical applications including the delivery of therapeutics for cancer therapy [12], tissue repair [13], or neurodegeneration treatments [14], as well as nucleic acid delivery for gene therapy [15].

The versatility of ION surface coatings has also promoted the selective magnetic labeling and tracking of cells within co-culture [16] or in vivo [17] studies. In particular, they have been employed as contrast agents for magnetic resonance imaging (MRI) [18,19], since their notable paramagnetic behavior grants superior transverse relaxivity (T_2_) than that of surrounding tissues and, in turn, creates a negative image contrast where they are located [20]. Besides medical imaging, the exceptional magnetization of functionalized IONs has allowed the magnetic separation of specimens (e.g., bacteria, viruses, macromolecules, cancer cells) for disease diagnosis [21] and the controlled migration [22] and sorting [23] of labeled cells both in vitro and in vivo. Hyperthermia treatments have also been broadly explored with these nanoparticles due to their high susceptibility to alternating magnetic fields to promote tumor cell apoptosis in cancer therapy, mainly due to thermal energy dissipation [24].

Due to these features, IONs are being widely studied for translational medicine applications and are an emerging commercial focus for numerous biomedical approaches. However, an efficient and controlled cellular internalization, as well as a stable intracellular accumulation of IONs, are major challenges for achieving superior performance. Understanding the mechanisms by which functionalized IONs interact with extracellular and intracellular environments is a key feature for the development of next-generation nanoparticles for highly specific functions. This review outlines the different aspects that govern ION endocytic uptake and subsequent escape from the endolysosomal pathway. Additional aspects affecting ION transfection are also included since many IONs require nuclear targeting for proper intracellular functionality. As such, we elucidate how IONs can be strategically tuned to induce an efficient uptake through the different endocytic pathways, avoid their early degradation due to lysosomal entrapment, and direct their intracellular fate.

## 2. Enhancing ION Internalization

The internalization of extracellular molecules is an essential process for cellular function and survival, but it is also a potential route of exposure to harmful agents. Endocytosis, as the most versatile and dynamic internalization mechanism, mediates the ongoing exchange between the extracellular and intracellular environments without disrupting intracellular homeostasis [25]. Due to the broad range of molecules that constantly undergo this internalization process, endocytosis provides continuous access to the intracellular space and it can be used to direct nanoparticle uptake. IONs can be strategically modified to be recognized, not only by specialized endocytic receptors that are known to internalize exogenous molecules but by almost any membrane component that undergoes endocytosis. Hijacking the endocytic machinery is, therefore, the most promising alternative for an efficient internalization of IONs and, coupled with strategies to escape intracellular degradation, represents an effective mechanism to enhance intracellular delivery. The different endocytic mechanisms involved during regular cellular uptake are discussed within this section, followed by the broad range of surface modification alternatives that can be used to internalize IONs through them.

### 2.1. An Overview of the Endocytic Mechanisms

**Figure 1:**
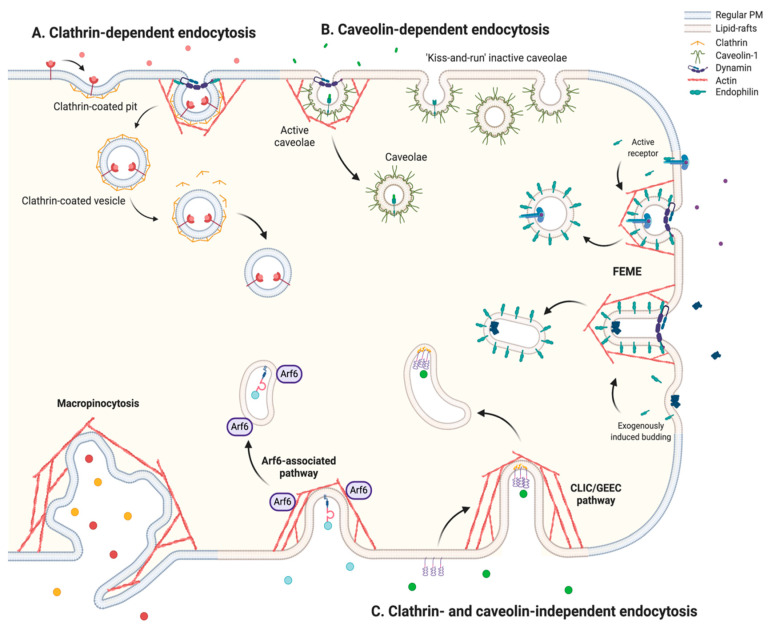
General schematic of the different endocytic mechanisms by pinocytosis, classified as (**A**) clathrin-dependent, (**B**) caveolin-dependent and (**C**) clathrin- and caveolin-independent (FEME, CLIC/GEEC pathway, Arf6 and macropinocytosis). (Created with BioRender.com).

Endocytosis can be broadly subdivided into two large categories, namely, phagocytosis and pinocytosis. Phagocytosis, commonly referred to as “cellular eating”, is a regulated process that comprises the ingestion of relatively large extracellular particles (>0.5 µm) intended for degradation, which is triggered by their interaction with specific cell receptors [26,27]. Phagocytosis is, therefore, the designated function of specialized Phagocytic cells of the immune system, since it allows the engulfment of invasive microorganisms, and can also aid in the elimination of cellular debris such as apoptotic bodies from tissues [28]. Conversely, pinocytosis mediates the controlled uptake of smaller molecules suspended in the extracellular fluid through invaginations of the plasma membrane (PM) and, in turn, is commonly referred to as “cellular drinking” [29]. This mechanism is responsible for the majority of cellular uptake [25]. Considering that only certain cell types are able to perform phagocytosis [30], and that typical ION size is below the normal phagocytic range, we will only focus on studying pinocytic mechanisms for internalization (Figure 1).

#### 2.1.1. Clathrin-Dependent Endocytosis

Overcoming the intrinsic tension of the lipid bilayer in a controlled manner is a crucial step towards membrane budding. Because this is a highly entropic process, it requires the aid of several scaffolding or destabilizing agents that disrupt the ordered and compact structure of the membrane [31,32]. Membrane budding is most commonly aided by clathrin, a trimeric protein that coats the cytosolic side of the membrane and guides its controlled invagination by creating lattice structures with different curvatures via polymerization [33,34]. The most remarkable feature of clathrin-mediated endocytosis (CME) is that the coating machinery can readily interact, through adaptor proteins, with a wide variety of transmembrane molecules that mediate the crosstalk with external agents ready to be endocytosed. Clathrin-coated pit (CCP) formation in cargo enriched regions is achieved through a complex and highly ordered process involving over 50 different accessory proteins that coordinate sequential clathrin recruitment, clathrin polymerization, and actin nucleation at internalization sites to eventually induce and stabilize the membrane invagination (Figure 1A) [35,36,37]. Vesicle fission is then coordinated by the recruitment of dynamin, a GTPase that favors vesicle detachment from the PM [38]. Accordingly, clathrin lattice structures allow the formation of spherical vesicles that range between 60 and 120 nm in diameter and, once internalized, lose their clathrin-coat and continue as naked vesicles [35].

CME is the most common endocytic mechanism in all cell types and tissues due to its high availability and adaptability towards recognizing numerous agents. Transmembrane receptors, for instance, can be recognized by accessory proteins of the CME machinery via short amino acid sequences in their structure or posttranslational modifications that arise upon ligand binding (e.g., phosphorylation or ubiquitination) [39,40]. Some of the most common receptors internalized through CME include transferrin receptors (TFRs), the low-density lipoprotein receptor (LDLR) family, insulin receptor (IR), receptor tyrosine kinases (RTKs) [41,42] and G-protein coupled receptors (GPCRs) [43]. Accordingly, a broad range of membrane proteins contain at least one of the many sorting determinants recognized by the CME machinery, which makes it a major contributor to receptor endocytosis dynamics [44]. As a result, targeting these receptors might be an attractive avenue for enhancing nanoparticle internalization (further discussed in Section 2.2).

#### 2.1.2. Caveolin-Dependent Endocytosis

Several cholesterol-dependent endocytic routes arise from lipid-raft regions of the PM, which are rich in cholesterol and glycosphingolipid microdomains [45]. Among the clathrin-independent and cholesterol-dependent mechanisms for endocytosis, caveolae are the most recurrent, especially in endothelial cells, fibroblasts, smooth muscle, and adipocytes [46]. Caveolae emerge from the interaction of caveolin, a membrane-spanning hairpin-like protein, with cholesterol in lipid-raft microdomains. Upon binding to lipid bilayers, caveolin promotes cholesterol clustering and is able to form higher-order hetero-oligomeric complexes that induce the positive membrane curvature and yield vesicular structures of 50 to 80 nm in diameter [47,48,49]. However, unlike the ubiquitous CCPs, dramatic differences have been observed for caveolar densities within different cell types and tissues [50] and, therefore, their contribution to endocytic dynamics varies widely. Unlike clathrin-coated vesicles, caveolae are not formed de novo in the PM, but instead, are readily available to endocytose cargo and detach upon activation [51,52]. In the absence of cargo stimulus, caveolae remain inactive near the PM and undergo continuous cycles of transient fusion and fission with it, a behavior that has been termed “kiss-and-run” [53]. However, the accumulation of activated receptors or the direct binding of ligands to caveolar regions can promote downstream signaling events (e.g., caveolin phosphorylation) that induce caveolar detachment and subsequent internalization (Figure 1B) [49,54]. Enriched receptors within caveolar regions vary significantly depending on the tissue and cell function, but insulin, albumin, and growth factor receptor-mediated phosphorylation of caveolin are some of the most observed caveolae activators [55,56]. The cellular entry of several viral vectors has also been reported to occur upon their interaction with integrins [57] or gangliosides (e.g., GM1, GM3) [58,59] within caveolar regions. Similarly, the binding of Cholera and Shiga toxins within caveolar regions proceeds by interactions with the GM1 and Gb3 glycolipids, respectively, thereby leading to local lipid rearrangement and glycolipid clustering, which have been shown to induce caveolae activation [60,61]. These pathogens feature clear examples of how this endocytic mechanism can be exploited without the need of transmembrane receptors.

#### 2.1.3. Clathrin- and Caveolin-Independent Endocytosis

Although CME and caveolae are the principal endocytic pathways, other cholesterol-dependent mechanisms have been identified as capable of internalizing cargo. Endophilin, which was originally identified as an accessory protein of CME involved in membrane destabilization and dynamin recruitment, was recently shown to be a crucial component for guiding the internalization of several RTKs, GPCRs and interleukin receptors, upon ligand activation, in a clathrin- and caveolin-independent manner [62,63,64,65]. This protein guides the formation of vesicles between 50 and 100 nm in diameter when coupled with the sequential action of the inward and outward forces created by the actin polymerization machinery [66,67,68]. This process is commonly termed as fast endophilin-mediated endocytosis (FEME) (Figure 1C) [69]. However, despite mainly relying on receptor activation, FEME also facilitates the uptake of Cholera and Shiga toxins upon their characteristic glycolipid clustering [67].

The endocytosis of glycosylphosphatidylinositol-anchored proteins (GPI-APs), unlike FEME, occurs irrespective of ligand binding. GPI-APs comprise a set of proteins that serve as receptors for numerous cellular processes and can be found linked to the extracellular leaflet of the PM through glycosyl bonds with phosphatidylinositol. They are laterally-organized in lipid-raft microdomains with cholesterol-induced clustering capabilities to induce membrane budding [70]. Due to the absence of coat proteins and dynamin independence, the resulting GPI-AP enriched vehicles are predominantly tubular structures of narrow diameter (~40 nm) but variable elongation [71,72]. This route is commonly referred to as the CLIC/GEEC pathway [73] (Figure 1C) and among its many functions, it favors the internalization of proteins involved in membrane repair (e.g., dysferlin), extracellular interactions (e.g., CD44) and cell motility (e.g., CD90) [74,75]. Additionally, it is specifically recognized for its implication in mediating the uptake of folic acid through the folate receptor [76].

Another clathrin- and caveolin-independent internalization mechanism is the Arf6-associated pathway, which modulates the constitutive internalization and recycling of certain GPI-APs and transmembrane proteins involved in membrane interactions with the extracellular environment (Figure 1C). The GTPase Arf6 supplies the local microenvironment of these receptors with the necessary elements for posterior membrane budding by mediating their recycling from previously internalized vesicles [77]. Most importantly, it favors the formation of coat-independent and dynamin-independent tubulovesicular structures of 60 to 200 nm in diameter [78,79]. Of particular importance is the role of this pathway in modulating the migratory phenotype of many cells [80] and favoring the continuous replacement of molecules involved in the regulation of extracellular matrix interactions [81,82], immune responses [83,84], and complement system regulation [85,86].

One last endocytic mechanism that has been reported to be clathrin-, caveolin- and dynamin-independent and that usually occurs in highly ruffled regions of the PM [87] is macropinocytosis (Figure 1C). This mechanism comprises the non-selective uptake of large volumes of extracellular fluid where molecules near the cell surface and present in the engulfed bulk fluid can be internalized [88]. To achieve this, actin-driven membrane extensions enclose an extracellular region and ultimately collapse and fuse with themselves or the cell surface [89]. The resulting vacuolar structure is called the macropinosome, which exhibits no apparent coating structure and is usually larger than 200 nm in diameter [90]. In turn, macropinocytosis readily provides an efficient, non-specific sampling of the extracellular environment that may contribute to the regulation of cellular dynamics [91,92].

### 2.2. Tuning IONs for Internalization

Due to the enormous versatility of the endocytic pathways, numerous surface modification alternatives arise for enhancing ION internalization. In particular, several strategies have emerged to promote the interplay between IONs and the PM. The interactions between these elements can be either adsorptive, due to nonspecific electrostatic interactions with the membrane, or receptor-mediated, due to nanoparticle recognition by specific receptors expressed in the cell surface. In both cases, ION-PM interactions promote the recruitment of the endocytic machinery from either of the previously described internalization mechanisms to facilitate ION internalization. Several surface modification strategies for enhancing ION internalization through adsorptive and receptor-mediated interactions are discussed below.

#### 2.2.1. Nonspecific Adsorptive Interactions

Modifying ION surfaces with biocompatible coatings has become a general consideration for their use in biomedical applications by considering that the large arsenal of available coating materials facilitates tuning the properties of ION-based systems to make them very versatile. The chemical composition of these coatings, for example, can be strategically selected to include relevant functional groups for posterior drug loading or the complexation of genetic material [93]. Physical properties, such as their surface charge or steric hindrance, can also be exploited for preventing Van der Waals- or magnetically-induced nanoparticle aggregation [94]. This is particularly important considering that pinocytic vehicles fail to internalize aggregates larger than 200 nm in diameter efficiently. However, beyond its benefits for nanoparticle architecture (i.e., topology and morphology), the coating selection has been commonly directed towards promoting endocytosis of IONs by their interaction with cell membranes. In this regard, it has been established that nanoparticle surface chemistry, especially surface charge, directly contributes to ION internalization through electrostatic interactions with the PM [95]. These interactions usually induce local lipid rearrangements, similar to those observed in toxin and viral uptake, that generally alter the local membrane curvature. Consequently, ION nonspecific adsorption to the cell surface due to coating charge has been proven to induce the activation of several endocytic mechanisms and promote ION internalization [96].

Nonetheless, due to their nonspecific binding to charged membrane components encountered in all cell types, internalization through adsorptive mechanisms are usually employed in applications where uptake specificity is not required. Cell labeling [97] and cell transfection [98] in vitro are the most common applications as they can be performed regardless of the cell type. Moreover, the resulting modified cells can be later included within tissue or disease models in vitro or be injected into in vivo models [22,99]. Similarly, due to the active role of immune cells and the reticuloendothelial system in the rapid clearance of nanoparticles from blood circulation, charged IONs can also be used for in vivo labeling and imaging of macrophages and tissues implicated in their clearance. This includes organs such as the liver, spleen, and lymph nodes where labeled IONs can be used to assess their functionality or detect inflammatory responses [100,101].

Accordingly, different considerations for enhancing ION internalization through nonspecific adsorptive interactions with the PM are discussed below. Because several reviews have already addressed the optimal nanoparticle size (10–100 nm) and shape (elliptical to spherical) for enhancing internalization [95,96,102], we will focus on reviewing the effects of surface chemistry on spherical ION uptake.

##### Cationic Coatings

**Figure 2:**
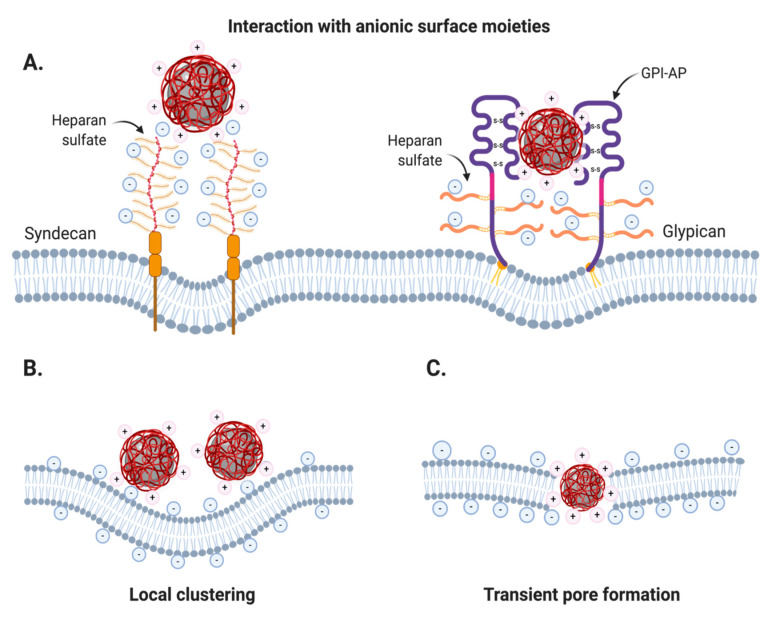
Adsorptive interactions between charged nanoparticles (CNPs) and the plasma membrane. (**A**) Electrostatic interactions with anionic syndecans and glypicans rich in heparan sulfate. (**B**) Cooperative membrane wrapping phenomena by cumulative CNP interactions with anionic phospholipids. (**C**) Transient pore formation by small CNPs (≤20 nm) due to strong attraction to the inner membrane layer in phosphatidylserine-rich regions. (Created with BioRender.com).

The surface charge contributes enormously to nanoparticle interactions with the PM due to the presence of a myriad of charged membrane components such as proteins, glycolipids and phospholipids. Cationic nanoparticles (CNPs), in particular, tend to be strongly attracted to the surface of PM due to negatively charged moieties in phospholipids and several membrane proteins [95]. Compelling evidence has suggested that sulfonated and carboxylated groups from many proteoglycans are targeted by CNPs and are closely implicated in their endocytosis (Figure 2A). In fact, due to their high negative charge, heparin/heparan sulfate proteoglycans, and to a lower extent, chondroitin sulfate B proteoglycans were shown to be major contributors for CNP endocytosis [103,104]. These proteoglycans commonly referred to as syndecans, tend to cluster upon multivalent binding of CNPs and associate with actin-binding proteins or F-actin to initiate endocytosis, which can proceed through clathrin-dependent and -independent mechanisms [105]. Heparan sulfate and chondroitin sulfate may also be linked to glypicans, which are extrinsic membrane proteins that are GPI-anchored and can mediate their endocytosis through lipid raft-dependent mechanisms [105,106]. However, the nonspecific binding of CNPs to the PM also promotes direct electrostatic interactions with phospholipids that induce membrane-wrapping phenomena. This is because polycationic coatings favor the interaction of IONs with several phospholipids at once to promote their local clustering and ultimately PM bending [107] (Figure 2B). Remarkably, experimental observations and dissipative particle dynamics simulations performed by Li and colleagues suggested that the local membrane curvature induced by a single nanoparticle is not enough for bud formation but, instead, a counterintuitive cooperative behavior between like-charged nanoparticles is needed [108]. Their agglomeration in the cell surface is responsible for initiating bud formation, which can later be aided by the regular endocytic machinery. The electrostatic interactions of CNPs smaller than 20 nm in diameter with the PM can also cause the formation of transient pores that facilitate nanoparticle translocation towards the cytoplasm (Figure 2C) [109,110]. This behavior has been attributed to their strong attraction to the internal membrane layer, which usually has a higher content of negatively charged lipids (e.g., phosphatidylserine) [110,111]. The complementary effect of these three PM interactions together accounts for the exceptional internalization efficiency of CNPs in most tissues.

Several polycationic molecules have been extensively studied for facilitating ION uptake, including synthetic and natural polymers, amphipathic lipids with cationic head groups, and cationic cell-penetrating peptides (CPPs). As shown in Table 1, a rationale for linking specific types of coatings with a particular endocytic mechanism is still missing, however, it is believed that factors such as nanoparticle size and differences in endocytic frequencies between cell types play a major role on their uptake dynamics [95]. In fact, several uptake mechanisms often contribute simultaneously, especially with nanoparticles that interact more avidly with membranes due to their increased charge density. However, although uptake efficiency increases with charge density, nanoparticle cytotoxicity increases accordingly [112,113]. Phosphatidylserine translocation from the inner to the outer layer of the PM has been observed during interactions with highly charged CNPs, presumably due to the strong attractive forces generated [114]. This behavior is a physiological hallmark for cell apoptosis and early necrotic-like cell damage and consequently, it can ultimately induce cell death [115]. Unconventional entry mechanisms influenced by high charge density have also been reported through further disruption of pre-existing membrane defects (e.g., membrane thinning, holes, or erosions). This has been thought to proceed by electrostatic interactions [116]. Increasing charge density directly augments CNP translocation frequency and allows the translocation of larger CNPs through non-endocytic mechanisms such as pore formation. Membrane permeabilization and enzymatic leakage are, therefore, commonly reported after exposure to highly polycationic nanoparticles [117]. However, the overall attractive features of CNPs have spurred a number of nanoparticle engineering initiatives intended to create potent cell-penetrating vehicles that avoid the reduction of cell viability [116].

The cytotoxic effect of the charge is more prominent in polycationic polymers than amphipathic lipids and CPP-conjugated coatings mainly because their charged groups are homogeneously distributed throughout the coating and are not just superficial. Although some studies have taken advantage of the high surface charge of polymeric coatings for inducing cytotoxic effects in cancer cells [118], a variety of strategies have emerged to counter their inherent charge density. A recent study by Sharkey and colleagues showed that when coating IONs with diethylaminoethyl-dextran (DEAE-DEX), an optimal ratio of 1:4 DEAE-DEX to IONs should be pursued to enhance nanoparticle uptake over time without increasing cytotoxicity [100]. An additional strategy has considered the conjugation of low molecular weight polymers to decrease the amount of condensed material on ION surfaces and, in turn, their net surface charge [119]. Chertok and colleagues, for example, coated IONs with a gum arabic polysaccharide matrix and subsequently conjugated low molecular weight polyethyleneimine (PEI) to create stable PEI drug delivery vehicles with no observable cytotoxicity [120]. Alternatively, Liu and colleagues developed an amphiphilic alkyl-modified low molecular weight PEI/ION nanoprobe for in vitro stem cell labeling that, instead of coating IONs directly, encapsulates ION clusters within micellar nanostructures [121].

Another report later showed that lactosylation of the N-alkyl-2kDa PEI/ION nanostructures maintained low cytotoxicity values at higher dosages without compromising labeling efficacy and MRI capability [122]. In addition, grafting and copolymeric configurations of polycationic polymers with neutral hydrophilic polymers (e.g., poly(ethylene glycol) (PEG)) have also been suitable alternatives for partially reducing charge density and stabilizing ION coatings [123,124,125,126]. This partial attenuation of charge proved efficient in maintaining the appealing effects of such polymers. 

As a result, with the proper considerations, cationic coatings have shown remarkable potential for mediating nanoparticle uptake. Shahnaz and colleagues, for example, showed the low cytotoxicity internalization in vitro of thiolated-chitosan- and chitosan-coated IONs in human endothelial progenitor cells (EPCs). The achieved efficiencies were around 17-fold and 6-fold higher than those obtained with uncoated IONs [127]. Similarly, Kumar Mishra and colleagues demonstrated that coating IONs with two different concentrations of poly(L-lysine) (PLL) (1 and 1.5 µg/mL) yielded a 2.5-fold and 4-fold increase in intracellular iron content when compared with uncoated IONs. In both cases, the cellular viability remained above 97% [158]. Low molecular weight (1.2kDa) PEI-decorated poly(glycidyl methacrylate) (PGMA) nanospheres encapsulating IONs also showed that PEI-modification caused their rapid internalization by neural progenitor cells (PC12). The internalization appeared to proceed by clathrin- and caveolin-independent mechanisms with no observable toxicity, while unmodified PGMA nanospheres remained uninternalized after 3 days of incubation [132]. Moreover, amphipathic lipids with cationic head groups, such as 1,2-dioleoyl-3-trimethylammonium-propane (DOTAP), have also shown remarkable potential for ION uptake. Preiss and colleagues developed DOTAP-oleic acid-coated IONs, assembled through hydrophobic interactions between DOTAP and oleic acid, which demonstrated superior cellular uptake in HeLa cells compared with hybrid shells containing cationic and anionic lipids [159]. However, they showed that by incorporating anionic lipids the charge-induced cytotoxicity is significantly reduced. They also established that an optimal ratio of 75% DOTAP and 15% 1,2-distearoyl-sn-glycero-3-phosphoethanolamine-N-[methoxy(polyethylene glycol)-2000] (PEG-DSPE) achieved the best internalization results.

IONs with immobilized arginine- and lysine-rich CPPs have also shown promising endocytic uptake potential, despite their high tendency to translocate cell-membranes. Recent work by our group showed that polyetheramine (PEA)-coated IONs conjugated with Buforin II (BUF II), an arginine-rich peptide, are able to efficiently penetrate THP-1 cells through energy-dependent and -independent mechanisms while maintaining cell viability above 90% [160]. After one hour of exposure, approximately 75% of the nanobioconjugates were internalized and a colocalization with endosomal compartments of about 27% was observed, as calculated from the Pearson Correlation Coefficient. Similarly, BUF II conjugation to iron oxide/silver nanoparticles coated with (poly(2-dimethylamino)ethyl methacrylate) methyl chloride (pDMAEMA) and PEA, in our most recent work, demonstrated high internalization rates in neuroblastoma cells (SH-SY5Y) due to the combined action of BUF II and the polycationic coating pDMAEMA, as well as similar endosomal colocalization degree as previously synthesized nanobioconjugates [161]. PEG-coated IONs conjugated with BUF II and Frenatin 2.3S CPPs, independently, have also colocalized with endosomal compartments in lung carcinoma (A549), neuroblastoma (SH SY5Y), gastric adenocarcinoma (AGS) and breast adenocarcinoma (MDA) cells (unpublished results). After 2.5 h of exposure, the colocalization efficiencies approached 44%, 42%, 13%, 38% for the BUFII nanobioconjugates and 34%, 37%, 18%, 28% for those of Frenatin 2.3S, respectively. This has been commonly attributed to the charge-induced electrostatic attraction of CPPs to sulfonated glycoproteins, which are able to mediate their entry through endocytic mechanisms [162,163]. For instance, poly(maleic anhydride-alt-1-decene)-dimethylamino propylamine (PMAL)-coated IONs decorated with the low molecular weight CPP, protamine, showed enhanced endocytic uptake through proteoglycan-dependent binding. The internalization results were superior than PEI and commercially available Lipofectamine nanoparticles [144].

##### Anionic Coatings

Unlike CNPs, anionic nanoparticles (ANPs) fail to translocate the PM and are only internalized through endocytic mechanisms. Due to their repulsive interactions with most membrane components, they exhibit lower PM affinity and, therefore, lower internalization rates have been frequently reported when compared to their cationic counterparts [164]. Cationic chitosan nanoparticles showed significantly higher uptake than anionic carboxymethyl-chitosan nanoparticles with similar size and absolute values of zeta potential (indicative of charge level) in both phagocytic and non-phagocytic cells [165]. Despite being less efficient than CNPs, ANPs have still proven superior uptake rates than neutral nanoparticles [166,167]. In fact, a comparative study of carboxymethyl-dextran (CMD) coated IONs, with similar size and different carboxyl functionalization efficiencies, demonstrated that increasing negative surface charge led to significantly higher nanoparticle uptake in colorectal adenocarcinoma (Caco-2) cells [148]. Interestingly, they also showed by inhibiting the endocytic pathways, that the uptake of CMD-IONs with higher anionic surface charge (−48 mV and −25 mV) was mediated by macropinocytosis, clathrin-dependent, and caveolin-dependent mechanisms. In contrast, CMD-IONs with lower anionic surface charge (−9 mV) depended solely on macropinocytosis and caveolae. These results suggest that the negative charge density not only affects nanoparticle uptake but the relative contribution of endocytic routes towards overall internalization. In addition, it has also been proven that as opposed to high positive charge densities, the negative ones appear not as disruptive, and therefore, ANPs showed much less cytotoxicity [95].

**Figure 3:**
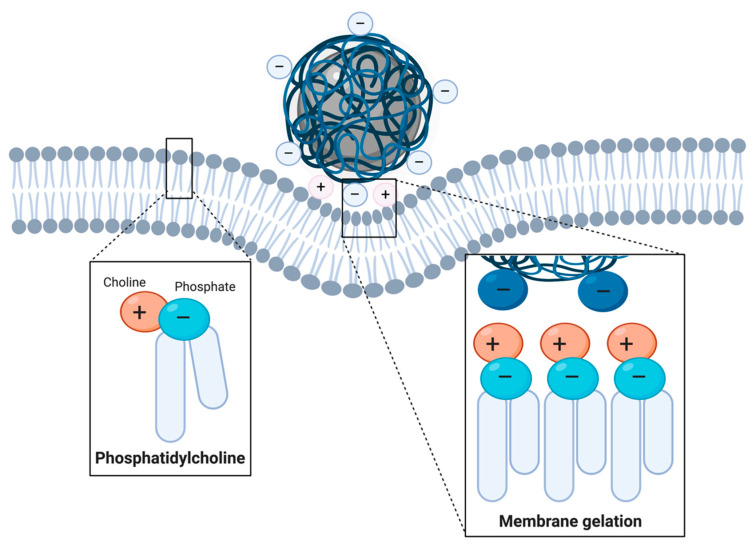
Local membrane gelation induced by ANPs in phosphatidylcholine-rich membrane microdomains. (Created with BioRender.com).

Although the precise mechanisms that govern the endocytosis of ANPs are not fully understood, several studies have suggested that their uptake takes place by promoting local changes in membrane properties. Wang and colleagues elucidated that the interactions of ANPs with PM may be more prone to occur in phosphatidylcholine-rich domains mainly because their head group is terminated by an electric dipole of phosphate and choline (P^−^–N^+^) [168]. ANPs can preferentially interact with the N^+^ terminus of phosphatidylcholine molecules by causing a slight tilt in their membrane position, which has been observed to transduce into local membrane gelation (Figure 3). Interestingly, coarse-grained molecular dynamics simulations have suggested that phase state changes induced by ANP adsorption are likely to induce stronger membrane curvature and nanoparticle wrapping than CNP adsorption. This is presumably due to the ANPs exerting higher lipid structural changes in such domains [169]. ANP-induced membrane budding can initiate endocytic processes and, as for CNPs, all endocytic routes are able to contribute to the internalization of polyanionic coated IONs (see Table 1). As such, several polymeric, lipidic, nucleic acid-based and inorganic coatings with inherent negative charge have been explored for ION uptake (Table 1).

In addition to their ability to interact with the PM, some receptors have been identified that can recognize ANPs through nonspecific interactions. Class A scavenger receptors (SR-As) are promiscuous transmembrane proteins that are able to interact with a wide variety of ligands in innate immune cells such as macrophages and dendritic cells. Moreover, they have also been observed in microglia, astrocytes, fibroblasts, endothelial and epithelial cells [170]. Due to their cysteine-rich binding sites, SR-As recognize polyanionic ligands through electrostatic interactions. This includes dimercaptosuccinic acid (DMSA) [152], carboxy-dextran [147], and dextran sulfate [149]. Novel PEG-coated IONs conjugated with thymidine-rich DNA oligonucleotides were recently developed for macrophage labeling and atherosclerotic plaque detection in vivo by exploiting their enhanced recognition by SR-As [146]. Strikingly, these nanoparticles demonstrated superior recognition by SR-As when compared to IONs conjugated with abasic thymidine-C3 spacer sequences of similar negative charges. This suggests that SR-As have a preferential affinity for oligonucleotides, which emphasizes the potential of nucleic acid coatings for enhancing ION internalization. Moreover, these receptors can be employed for the uptake of gene delivery vehicles with high nucleic acid loading [171]. As a result, SR-As provide an efficient internalization route for macrophage labeling or in vitro studies with selected cell lines, which partially explains the superior clearance of negatively charged nanoparticles in vivo [172].

##### Effects of Serum Protein Adsorption on Coated Surfaces

Charged surface coatings usually promote protein adsorption in biological fluids through interactions with serum proteins. This creates a protein corona on the nanoparticle surfaces that can alter physical properties such as size and charge (see Table 1) [157]. Some studies have suggested that protein adsorption may favor nanoparticle uptake by promoting their recognition by receptors that regularly internalize the adsorbed proteins. Bovine serum albumin (BSA), for example, was shown to bind to ANPs and promote their uptake through the albumin receptor in green monkey kidney epithelial cells (BS-C-1). Alternatively, BSA binding to CNPs promoted their uptake through SR-As due to albumin denaturation upon adsorption [173]. However, other studies have reported that serum protein adsorption, both in vitro and in vivo, may decrease ION uptake due to attenuation of surface charge and increased nanoparticle agglomeration [153,174]. These adsorbed proteins have also shown to be major contributors to their in vivo recognition by macrophages and the reticuloendothelial system through opsonin receptors. Upon recognition of opsonins (e.g., complement proteins, immunoglobulins, apolipoproteins) adsorbed on the nanoparticles, they are phagocytosed [175].

This evidence suggests that protein adsorption should be closely monitored during nanoparticle engineering to adequately assess their performance in physiologically relevant media. Although the proteins adsorbed depend on nanoparticle surface charge [176], Sukulkhu and colleagues demonstrated that the chemical composition of their coating plays a major role in protein adsorption [177]. For this reason, this should be a major consideration during coat selection (see [178] for further details). A common approach for addressing protein fouling is incorporating neutral polymers to generate repulsive steric forces that ultimately interfere with protein adsorption. Examples of such polymers include PEG, dextran, and poly(vinyl alcohol) (PVA) [179,180], however, the absence of surface charge usually leads to a reduction in nanoparticle uptake, as shown before. Optimizing surface interactions with the PM while controlling nonspecific protein adsorption is, therefore, one of the major challenges during nanoparticle development for translational applications

#### 2.2.2. Receptor-Mediated Interactions: Targeted Internalization of IONs

Although adsorptive interactions between charged nanoparticles with the PM grant promising internalization outcomes, their nonspecific uptake limits their implementation in applications that require specific cell targeting. Targeting specificity is particularly important for applications that involve disease diagnostics through medical imaging or the delivery of therapeutics in vivo as such applications require nanoparticle accumulation in specific tissues [181]. Targeted delivery of nanoparticles to specific tissues is straightforwardly addressed by modifications of coated surfaces with agents that are specifically recognized by receptors in such tissues. These agents should be selected according to the expression profile of membrane receptors in the target tissue. Their relative expression with respect to surrounding tissues should be assessed as these should be predominant for adequate selectivity. Accordingly, nanoparticle recognition by these receptors not only guarantees tissue selectivity but also serves as a direct entry route into cells by receptor-mediated endocytosis.

Due to the broad spectrum of cell surface receptors that can be targeted for nanoparticle endocytosis, virtually any tissue could be suitable for delivery with the proper engineering of the nanoparticle architecture. However, some tissues are more easily targeted than others due to expression differences in the receptors of interest. ION delivery to carcinogenic tissues and across the highly regulated blood-brain barrier (BBB) are discussed below to exemplify targeted internalization through receptor-mediated endocytosis.

##### Uptake in Carcinogenic Cells

ION systems have been widely employed for cancer theranostic applications due to their promising potential as MRI contrast agents for cancer diagnosis, drug delivery vehicles for chemotherapy, and intracellular hyperthermia treatments [182]. Moreover, due to the marked phenotype differences between carcinogenic and healthy cells, overexpressed receptors in cancer cells serve as exceptional targets for targeted delivery of IONs. The transferrin receptor (TFR), for example, is overexpressed in numerous tumor cells, most notably in breast cancer [183]. As one of the most studied CME ligands, transferrin has been commonly used as a targeting agent and conjugated to numerous nanocarriers [184,185]. For instance, Gharib and colleagues developed magnetic nanoliposomes composed of dipalmitoyl phosphatidylcholine (DPPC), distearoyl phosphatidylcholine (DSPC), cholesterol, and loaded with IONs. The formulation also included transferrin and artemisinin (anti-cancer drug) and was administered intravenously to breast cancer-bearing BALB/c mice [186]. Compared to free transferrin and free artemisinin administration, this loaded nanocarrier led to a 5.5- and 10-fold increase in their accumulation, respectively. Moreover, when guided by an external magnetic field, their concentration was 3.8- and 4-fold higher than in its absence. Importantly, they not only inhibited primary tumor growth, but they also reduced tumor volume in tumorized mice 15 days after treatment. Similarly, Wang and colleagues demonstrated that the in vitro uptake of DOX-loaded, transferrin-conjugated chitosan-IONs in human brain tumor (U251) cells was around 8 times higher than that of DOX/chitosan-IONs and yielded a 2-fold increase in apoptosis rate [187].

Due to the invasive phenotype and high angiogenic activity of cancer cells, the epidermal growth factor receptor (EGFR) and vascular endothelial growth factor receptor (VEGFR) are commonly overexpressed in tumor tissues and, therefore, are frequent targets for cancer therapies. Although IONs with conjugated physiological ligands for these receptors have been developed [188,189], humanized antibodies are preferred to avoid activation of the respective signaling pathways without detrimentally impacting the constitutive endocytosis by CME. The most common antibodies for EGFR targeting include cetuximab [190], nimotuzumab [191], and single-chain antibody fragments (ScFv) [192]. In the case of VEGFR targeting, the preferred antibodies include bevacizumab [193] and anti-VEGF monoclonal antibodies [194]. The human epidermal receptor 2 (HER-2), a member of the EGFR family, has also been targeted with ION modified with a number of antibodies, including trastuzumab [195] and the anti-HER2 affibody [196]. Although HER-2 has no natural ligands and is rarely endocytosed, HER-2 antibody binding induces post-translational modifications that mediate its internalization through CME [197].

GPI-APs such as the folate receptor (FR) and CD44 have also been frequently targeted due to their important roles in tumor growth and migration [198,199]. Ligands for the FR (e.g., folate) and CD44 (e.g., hyaluronic acid, CD44 antibodies) have been, therefore, widely used along with IONs for tumor targeting both in vitro and in vivo [200,201,202,203]. Although these receptors are commonly internalized through lipid raft-dependent mechanisms (e.g., CLIC/GEEC pathway), recent reports have also described the contribution of CME [204,205]. This is presumed to occur due to the high abundance of CME in all cell types, which can make them passive cargoes of CCPs [44], especially when nanoparticle multivalency increases [206]. Among other commonly overexpressed receptors, underglycosylated mucin 1 (uMUC1), membrane-bound matrix metalloproteinase (MMP-2), carbonic anhydrase IX (CA-IX), CD22 and α_v_β_3_ integrins have also been targeted with IONs tethered with various ligands. Examples for each receptor include the synthetic peptide EPPT1 [207], chlorotoxin [155], the monoclonal antibody M75 [208], anti-CD22 antibodies [209] and RGD peptides [210], respectively.

Overall, numerous receptors have been identified for cancer therapy (see Table 2) and, although most of them are overexpressed in all tumor cells, some can be more prominent within specific tumors. Consequently, it is particularly important to assess the physiology of the targeted cancer cell type for the adequate selection of targeting agents (see [211] for further details on cancer targeting).

##### Uptake by the BBB: Delivery to the Brain

Nanoparticle delivery to the brain has gathered special interest for the diagnosis and treatment of neurodegenerative diseases, cancer and other brain-derived pathologies. However, unlike the endothelial barriers between the blood and other tissues, transport across the BBB is much more regulated and poses an additional constraint for targeting neural tissues [231]. Although disease conditions are known to increase BBB permeability, diagnostic and therapeutic strategies are ideally performed at early stages before BBB disruption [231]. Therefore, nanoparticles should be able to cross healthy barriers. As such, several targeting agents have been identified that facilitate ION transcytosis through specialized brain microvascular endothelial cells (BMECs), which grant their access to the brain parenchyma (Table 2). The low-density lipoprotein receptor-related protein 1 (LRP1) is highly implicated in the transport of β-amyloid peptides, angiopep-2, and lactoferrin across BMECs and, therefore, has become a recurrent target for brain delivery [223,232]. As a member of the LDL receptor family, LRP1 is generally recognized by the CME machinery, although some studies have shown that caveolin-dependent endocytosis is also implicated [223,233]. Xin and colleagues demonstrated that PEG-poly(e-caprolactone) coated IONs and further conjugated with angiopep-2 were easily loaded with paclitaxel (anti-cancer drug) to efficiently cross an in vitro BBB model. The nanovehicles were found throughout the whole brain but preferentially accumulated in an intracranial glioma tumor after intravenous administration in U87-MG tumor-bearing mice [233]. In contrast, nanoparticles without angiopep-2 passively accumulated only in the tumor region because of tumor-induced BBB disruption. LRP1 overexpression in cancer accounted for this increased tumor accumulation and, in turn, angiopep-2, as well as lactoferrin, have shown exceptional results for brain tumor targeting [222,233].

Transferrin-conjugated IONs have also shown promising transport across the BBB and in vivo brain accumulation due to TFR enrichment in BMECs [215]. In particular, brain tumor targeting is one of their most common applications mainly due to the ease of recognition by cancer cells as well [184]. Although targeting of TFR has been achieved with the aid of antibodies such as OX26 [218] and RI7-217 [217], they have shown different affinities within animal models (e.g., rats vs. mice) [234]. Similarly, antibodies have been employed for targeting the insulin growth factor receptor (IGFR), which is also highly expressed in the BBB and taken up by CME [235]. For instance, Boguslaw and colleagues developed dextran-coated IONs functionalized with the anti-insulin-like-growth-factor binding protein 7 (anti-IGFBP7) single domain antibody, which effectively accumulated in the brain of glioma tumor-bearing mice. The nanovehicles mainly accumulated in the tumor and no nonspecific accumulation in other organs was observed [230].

## 3. Enhancing ION Endosomal Escape

After internalization, all endocytic vesicles eventually converge into a common endosomal pathway by fusing with a pleomorphic compartment, known as the early endosome (EE), responsible for the careful sorting of internalized cargo [66]. As the major general sorting station and crossroad for internalized molecules, EEs are highly dynamic and frequently undergo fusion with one another or incoming vesicles. Endocytosed vesicles are targeted to the EE by the intracellular conversion of phosphatidylinositol (PI) into PI3P and the acquisition of both rabenosyn-5 (Rab5) and early endosome antigen 1 (EEA1) markers [236], which ultimately mediate their fusion. Upon EE integration, the slightly acidic environment of EEs (~pH 6.2) facilitates the dissociation of ligand-receptor complexes and favors the segregation of membrane-bound elements according to their intracellular fate [237]. Constitutively internalized transmembrane receptors are either recycled directly towards the PM (fast recycling pathway) or directed towards perinuclear-localized recycling endosomes (REs), which regulate slow transport back to the PM (slow recycling pathway). Conversely, transmembrane receptors that are destined for degradation, labeled through ubiquitination, are removed from the surface of EEs by the formation of intraluminal vesicles (ILVs) to avoid their recycling. ILVs, in conjunction with luminal molecules, continue within EEs through their maturation into late endosomes (LEs), in which Rab5 is converted into Rab7 and the lumen is progressively acidified and enriched with lysosomal hydrolases [238]. LEs eventually deliver their luminal contents to lysosomes where the hydrolytic environment favors cargo breakdown [239].

Considering this scenario, endocytosed IONs are most likely to dissociate from vesicle membranes and reside within the acidic lumen of endosomes, which means that by default they will be targeted towards degradation. Therefore, if IONs are not intended for lysosomal treatments, they must escape endocytic vesicles to avoid degradation or recycling processes [240]. The nanoparticle-induced disruption of endocytic vesicles is known as endosomal escape, which is fundamental for the effectiveness of ION-based treatments intended to have therapeutic activity in the cytosol or the nucleus [241]. Accordingly, research has shown that endosomal escape is a rate-limiting step within the delivery of therapeutics to the desired intracellular locations [241,242]. The endosomal escape potential of IONs depends on how the surface is modified to induce different vesicle leakage pathways [240]. However, these mechanisms remain highly contentious due to the complexity of their analysis [243]. Among the proposed alternatives for targeting endosomal escape, this section will focus on the proton-sponge effect, passive diffusion, translocation and pore formation, membrane fusion, and photoinduced membrane disruption strategies.

### 3.1. Proton-Sponge Effect and Osmotic Lysis

**Figure 4:**
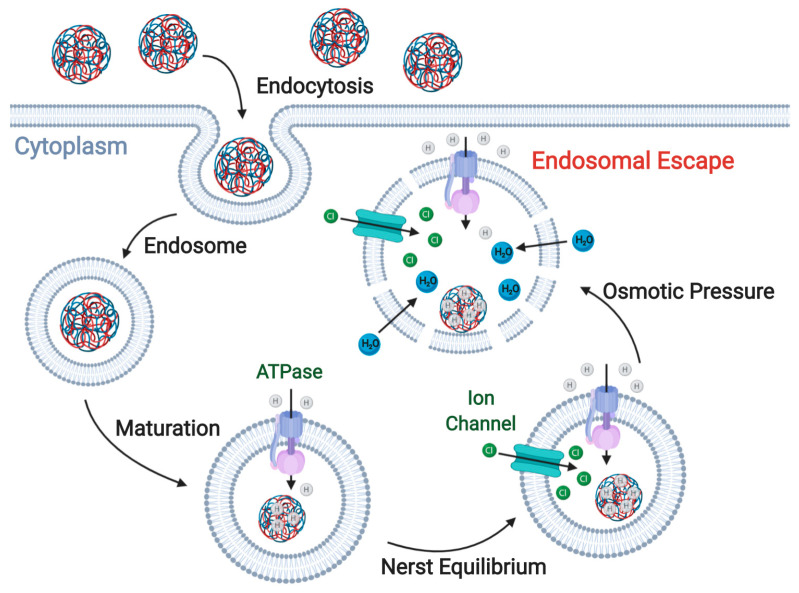
Endosomal escape mediated by the Proton-Sponge Effect. Surface coatings of IONs sequester protons from the endosomal lumen and create an osmotic gradient. The increase in osmotic pressure, coupled with destabilizing interactions between cationic surfaces of IONs and the endosomal membrane ultimately leads to the lysis of endosomal vesicles. (Created with BioRender.com).

The proton-sponge effect is one of the most common mechanisms for endosomal disruption, which results from an increased osmotic gradient and polymer swelling generated by the accumulation of buffering ionic molecules inside endosomes. ION surface modification with polyamines is commonly used to trigger this endosomal escape pathway [244,245]. After the endocytic uptake of exogenous molecules, early endosomes start their maturation process by an ATPase-mediated proton influx that drives endosomal acidification [244]. However, polyamines might act as buffers and sequester the incoming protons mainly because the pKa values of amine groups are in the range of the endolysosomal pH values. As a result, they are able to maintain a constant pH and alter the Nernst equilibrium potential. To restore such an equilibrium, an influx of chloride anions is generated through ion channels [245,246]. This is followed by their intraluminal accumulation, which creates an osmotic gradient that increases water influx and swells the endosome. In addition, polymer protonation creates internal electrostatic repulsion forces that increase its volume, which can lead to further swelling of endosomes and posterior membrane disruption. This phenomenon ultimately results in the release of all endosomal content into the cytoplasm (Figure 4). Recent reports show evidence that endosomal rupture occurs when the intraluminal volume increases by approximately 5% and is further enhanced by electrostatic interactions between the protonated polymers and the endosomal membrane [244]. To achieve an effective endosomal escape through the proton-sponge effect, an optimal balance is important between the osmotic pressure, the polymer swelling, and the degree of interaction between the polymer and the membrane. However, considering that the endosomal size and membrane leakiness may vary between cell types and tissues, it is important to establish experimentally the optimal combination of such parameters. Otherwise, it is possible to reach a sub-optimal operation regime where instead of a completely disrupted endosomal membrane, it just has minor defects and is barely leaky [247].

For non-lysosomal therapies to be effective, IONs are usually functionalized with materials that can confer the ability to escape endosomes by the proton-sponge effect. A widely used material is hyperbranched PEI, which in its liquid state contains approximately 30% primary, 40% secondary, and 30% tertiary amines. Although some of these pendant groups are charged at physiological pH, most of them can be protonated during luminal acidification, which makes PEI suitable for both non-specific adsorptive endocytosis (see Section 2.2.1) and inducing the proton-sponge effect [248]. In consequence, PEI has been extensively used to modify the surface of IONs, primarily for gene editing applications where endosomal escape is a fundamental step for an effective transfection and electrostatic interactions can be exploited to form complexes with nucleic acids [98,249,250]. In this regard, Rohiwal and colleagues developed CRISPR/Cas9-PEI-IONs capable of disrupting endosomes and effectively inserting the genes of both the blue fluorescent protein (BFP) and the green fluorescent protein (GFP) in vitro [251]. Similarly, pluronic/PEI shell crosslinked nanocapsules with embedded iron oxide nanocrystals (PPMCs) were developed by Lee and colleagues for the delivery of siRNA to cancerous cells. They showed that the vehicle effectively escaped endosomes and was able to successfully suppress GFP expression in vitro [249]. In another recent work, Steitz and colleagues developed colloidally stable ION-PEI-DNA beads at high salt concentrations over a wide pH range that enhanced endosomal escape, transfected COS cells, and showed lower cytotoxicity compared with PEI-DNA [252].

Water-soluble chitosan functionalization of IONs has also been reported to enhance endosomal escape by the proton-sponge effect and allows the subsequent long-term retention of the core IONs in vitro [97]. Given that the imidazole group of histidine has a pKa near 6.0, histidine-rich peptides can also act as a buffer to absorb protons and induce the proton-sponge effect. This approach was explored by Song and colleagues through novel PolyMag/DNA/Tat-peptide nanoparticles with endosomal escape abilities and a 4-fold improvement in transfection over the complexes without the peptide [253]. The same capability has also been shown for ION-PEA-BUF-II nanobioconjugates developed by our group, which were able to efficiently penetrate several mammalian cell lines without significant impact on viability and presented an overall homogeneous cytosolic distribution [131,254]. In addition to agents suitable for protonation, Cristofolini and colleagues developed a novel vehicle that induces the proton-sponge-effect by increasing the luminal concentration of cationic molecules to form an internal hypertonic medium. Caffeic acid–magnetic calcium phosphate (Caf-MCaP) nanoparticles were designed as gene carriers capable of escaping endosomes by increasing osmotic pressure as a consequence of the increased luminal concentration of calcium ions [255].

### 3.2. Membrane Translocation Mechanisms

**Figure 5:**
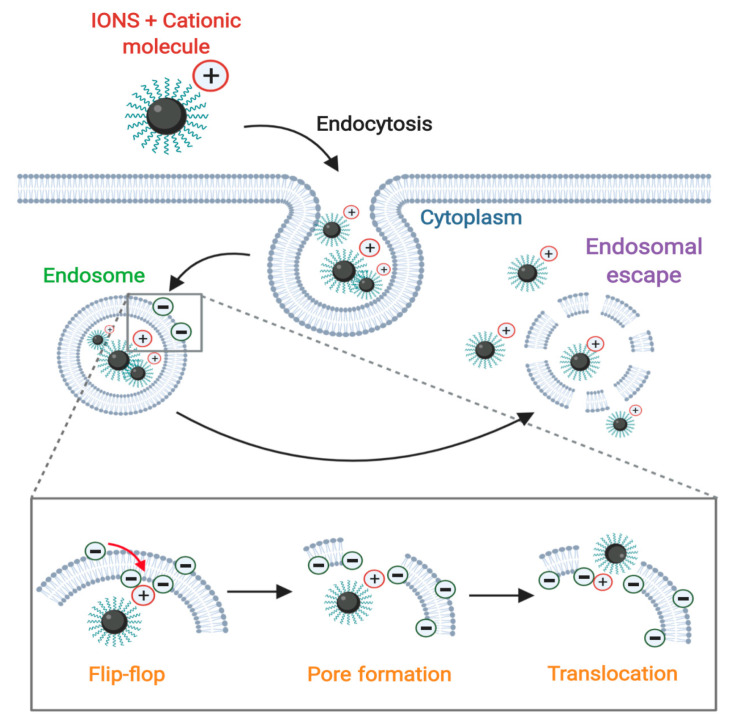
Schematic illustration of the translocation mechanism. Nanoparticles (IONS coated with cationic molecules) are endocytosed by the cell and internalized inside endosomes. The positive charge of the coated nanoparticles generates a flip-flop of the endosome’s cytosolic anionic lipids, which induces the generation of pores through which the nanoparticles can cross the endosomal membrane to reach the cytosol. (Created with BioRender.com).

Pore formation by translocation mechanisms on membrane vesicles can occur through cationic peptides or proteins derived from several viral, bacterial, vegetal, and animal sources [256]. Although pore formation mechanisms are poorly understood, the most common hypothesis claims that these molecules can induce pore formation when they self-assemble across the membrane of the endocytic vesicles [243]. This process occurs when these cationic molecules interact with anionic groups in the external face of the endosome, thereby generating a “flip-flop” structural change that leads to nanoscale disruptions in the membrane (Figure 5) [257]. Recent studies have shown that the translocating abilities of peptides and proteins are attributed to their content of arginine [256] and lysine [256,258], which confer cationic characteristics. These residues are involved in penetration and destabilization of membranes [256] via binding to the phosphate groups of the polar head of phospholipids, which locally destabilize the membrane and facilitate nanoparticle translocation [259].

The most commonly employed translocating peptides are those derived from viral organisms [260] because of their ability to mimic the endosomal disruptive characteristics of viral agents [256]. Among these, the HIV-1 transcriptional activator (TAT) peptide has been the most intensively studied [261]. Wang and colleagues, for example, showed that TAT coating of FITC-IONs significantly enhanced their cytosolic distribution, while the uncoated ones mostly remained trapped within endosomal compartments [261]. In fact, Nair and colleagues demonstrated, with the aid of 3D-electron tomography, that TAT-IONs slowly released from endocytic vesicles in human glioblastoma cells [260]. A similar study showed that aminated dextran-ION vehicles coated with a TAT-derived peptide escaped endosomes very effectively. This allowed them to track hematopoietic and neural progenitor cells in vivo [262]. Moreover, Hauser and colleagues exploited the endosomolytic potential of TAT-functionalized IONs to develop a synergistic cancer treatment that employs a combined biochemical and radiation therapy approach [263]. The developed nanobioconjugates efficiently escaped lysosomal vesicles and their cytosolic distribution improved radiation therapy by increasing reactive oxygen species (ROS) generation.

Another commonly used translocating peptide is poly-arginine [264]. Veiseh and colleagues demonstrated that ION–poly-arginine nanobioconjugates were 3-fold more potent in delivering siRNA due to their endosomal translocating abilities. Moreover, these nanobioconjugates were significantly less cytotoxic than uncoated IONs [265]. Similarly, the translocating peptide gH625, derived from the glycoprotein H of the Herpes simplex virus 1, has been used since it possesses a high content of arginine and lysine [266]. gH625-ION nanobioconjugates not only showed an effective pass of the blood-brain barrier but a homogeneous cytosolic accumulation in astrocytes and pericytes [266]. Moreover, Perillo and colleagues showed that gH625-PEG-IONs have high cytoplasmic distribution, making them good candidates for cancer theranostic treatments [161]. BUF II-conjugated IONs have also demonstrated great translocating abilities due to the high arginine content of BUF II, as we have shown in previous studies [131,132]. ION-PEA-BUF II and Ag/ION-pDMAEMA/PEA-BUF II nanovehicles showed homogeneous cytosolic distribution after 1 h of incubation with THP-1 and neuroblastoma (SH-SY5Y) cells, respectively, and only 27% and 24% colocalized with endosomal compartments after this time period [131,132].

Translocating proteins of bacterial origin are also known for penetrating endosomal membranes, and especially cell membrane surface proteins [23]. This has been mainly attributed to their involvement in membrane destabilization of phagosomes and in facilitating the release of bacteria into the cytosol of the host cells [241]. For instance, Sherwood and colleagues used IONs coated with binding compounds of bacterial membranes for the development of drug delivery systems with endosomal escape abilities, which can be potentially used for the delivery of pharmacological cargoes [267]. Similarly, in previous work by our group, we developed novel cell-penetrating nanobioconjugates by the immobilization of the outer membrane protein A (OmpA) of Escherichia coli on IONs. After 10 min of incubation, the obtained nanobioconjugates achieved endosomal escape levels above 25% in THP-1 cells, as estimated from their colocalization with endosomal compartments [268]. Recently, we also designed multifunctional orthopyridyl disulfide-PEG succimidyl ester (OPSS-PEG-NHS) coated IONs to enable the delivery of siRNA for silencing the BACE1 gene expression, as potential treatment of Alzheimer’s disease. The immobilization of OmpA on the surface of these PEGylated IONs increased their endosomal escape efficiency in neuroblastoma cells from 68% to 88%, demonstrating the potent escape abilities of this protein [269].

### 3.3. Membrane Fusion

**Figure 6:**
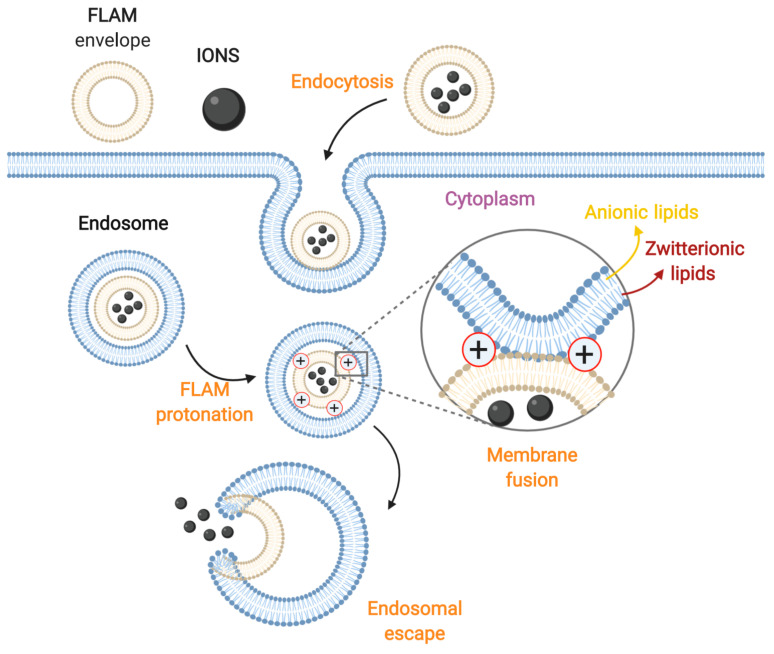
Schematic illustration of the membrane fusion mechanism of fusogenic lipids or amphiphilic molecules (FLAM)-ION complexes. The nanoparticles are encapsulated within a FLAM envelope for subsequent internalization by endocytosis. Within the endosome, the FLAM phospholipids protonate, thereby inducing the fusion of this envelope with the Zwitterionic luminal lipids of the endosomal vesicles. This process ultimately leads to endosomal escape. (Created with BioRender.com).

IONs assembled with fusogenic lipids or amphiphilic molecules (FLAM) can induce endosomal escape via fusion of the FLAM envelope with the endocytic membrane, which inverts its structure and allows the release of encapsulated cargoes into the cytosol (Figure 6) [243]. To exploit this penetration mechanism, FLAM-ION complexes must be first trapped into endosomes [257]. This is because the acidic pH inside endocytic vesicles might induce protonation and conformational changes in FLAM that eventually lead to local membrane destabilization. This process occurs via interactions between zwitterionic luminal lipids of endosomes and protonated FLAM structures and ultimately results in their fusion [243,257]. The most commonly prepared FLAM-ION complexes are IONs encapsulated in liposomes. These carriers are bilayered vesicles made of self-assembled amphiphilic phospholipids in aqueous solutions [270]. Liposomes are widely used due to their capacity to maintain the properties of nanoparticles, as well as to enhance their internalization and improve their stability in aqueous solutions [271]. Protonation of FLAM liposomes with anionic head groups weakens their amphiphilic micellar structure and has shown to cause their reorganization due to hydrophobic forces. The resulting structures interact more favorably with endosomal membranes through hydrophobic interactions to promote their fusion. This allows the release of encapsulated cargoes into the cytosol [243]. The incorporation of cholesterol within liposomal structures has shown to enhance their fusogenicity by increasing the contact sites needed for lipid-mixing and helping expand the fusion pore [272]. The addition of cationic lipids within FLAM liposomes also favors this outcome because electrostatic interactions further destabilize endosomal membranes and facilitate the fusion process (Figure 6) [257].

However, as mentioned above, charged surfaces of liposomes may increase their clearance by the reticuloendothelial system and reduce their circulation time prior to reaching target cells. As a result, liposomes modified with neutral hydrophilic PEG, known as PEGylated lipoplexes, have become an attractive alternative over the past few years [273,274]. PEG chain modifications generate amphiphilic micelles [274], which can maintain the colloidal properties of liposomes [275]. These modifications provide nanoparticles with a protected environment from undesirable reactions in the biological media [273], promote bioavailability, decrease immune genotoxicity [271], increase mean residence time in the bloodstream [275] and improve the cytoplasmic distribution of the nanoparticles [276]. Despite these benefits, PEGylation can also interfere with membrane fusion and destabilization dynamics by attenuating electrostatic interactions [277]. Consequently, an optimal balance between PEGylation degree and fusogenicity should be obtained when using PEGylated liposomes for nanoparticle delivery.

Recent studies have shown that PEGylated liposomes loaded with IONs enhance contrast efficiency for MRI. This is mainly because the formed lipoplexes increase the distribution of nanoconjugates in the cytosol of cells in the tissues of interest [273,275]. Hardiansyah and colleagues synthesized anionic magnetoliposomes with 19 mol% cholesterol and 4.8 mol% PEGylation that efficiently escaped endosomes and, combined with a magnetic field, successfully enabled the time-controlled release of cargoes [278]. In the same way, Amstad and colleagues demonstrated that palmityl-nitroDOPA coated IONs encapsulated in 5 mol% PEGylated zwitterionic liposomes internalized effectively and escaped endosomes. Moreover, they maintained a relatively high magnetic susceptibility, which made them attractive candidates for cellular time-dependent treatments [279]. Additionally, Cardoso and colleagues demonstrated that with only 5 mol% PEGylated zwitterionic phospholipids, the obtained magnetoliposomes increased circulation time and maintained the fusogenic properties [280].

### 3.4. pH-Triggered Endosomal Escape

**Figure 7:**
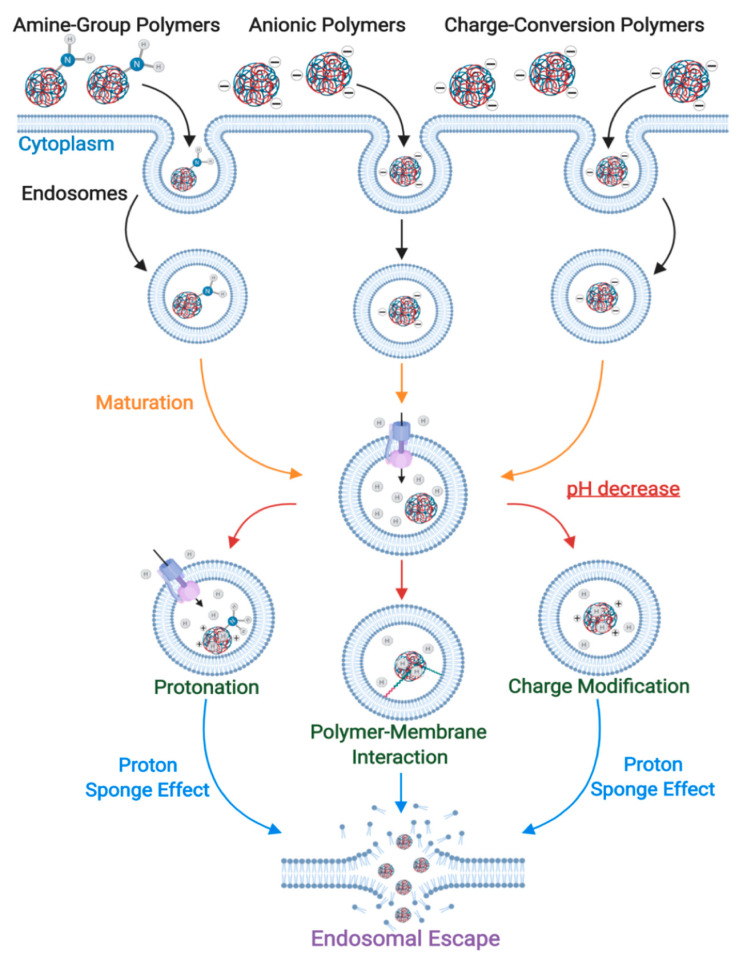
pH-triggered endosomal escape strategies via polymers susceptible to protonation. This includes polymers with pendant uncharged amino-groups at physiological pH, anionic polymers and charge-conversion polymers (Created with BioRender.com).

pH-responsive delivery systems have also been developed to enhance endosomal escape without affecting the performance of nanoparticle-based vehicles. Given the pH dependence of the maturation process of endosomes, these systems can be tuned to respond to pH changes and enable the escape of immobilized cargoes to the cytosol [281]. Although polyamines with moieties of different pKa values can enhance endosomal escape through the proton-sponge effect (e.g., PEI), these are usually cationic at physiological pH and their use is limited by their inherent cytotoxicity. Alternatively, pH-sensitive polymers can be used as coatings to reduce the toxicity of nanoparticles while responding to the acidification processes by protonating or degrading. This process usually leads to disruption of the membrane and endosomal escape (Figure 7). Amine-rich polymers with pKa values below physiological pH have attracted attention due to their ability to protonate and induce the proton-sponge effect. Accordingly, polymers rich in imidazole groups (pKa ~6.0) are among the most exploited for pH-responsive ION surface modifications [253]. Charge-conversion polymers have also emerged as a plausible alternative as they exhibit a net negative charge at physiological pH but become positively charged upon interactions with the acidic medium of endosomes [240]. These polymers show this mixed behavior due to the presence of both acidic and basic functional groups, which change their protonation status during endosomal acidification. Rahman and colleagues developed N-itacolynated chitosan-coated IONs crosslinked with ethylene glycol diglycidyl ether (NICS-EGDE-IONs), which had both carboxylate and amine moieties, and demonstrated high endosomal escape mainly due to their capacity to induce the proton-sponge effect in acidic environments [282]. Similarly, polycations conjugated with anionic moieties through acid-cleavable linkages exhibit response to endosomal acidification. In such an environment, the external anionic layer degrades and enhances endosomolytic electrostatic interactions, in addition to the proton-sponge effect [283,284]. Alternatively, anionic pH-sensitive polymers containing weak acidic groups and hydrophobic moieties (e.g., poly(acrylic acid)-derivatives, poly(malic acid)-derivatives and L-phenylalanine-grafted poly(L-lysine iso-phthalamide)) can also be exploited due to their membrane disruptive capabilities upon luminal acidification [285,286,287,288]. In this regard, at pH values below their pKa, the polymers undergo a conformational change from an extended charged configuration to a globular hydrophobic one. This change facilitates the intercalation of polymer chains within the hydrophobic regions of endosomal membranes to disrupt their ordered structure [289].

Liposomes have also been prepared with pH-responsiveness to enhance endosomal escape even further. This not only favors fusion with endosomal membranes by protonation of the anionic head groups of phospholipids but the ability to further destabilize them via pH changes. For instance, several studies have incorporated pH-responsive polymers on liposomal surfaces or inside liposomes, which facilitate cargo release by destabilizing liposomal and endosomal membranes [290,291]. Similar fusion events are observed when glutamate-rich fusogenic peptides (e.g., GALA, pHLIP, INF7) are incorporated, mainly because their amphipathic structures switch from random coil to α-helical upon pH-triggered glutamate protonation [292,293,294]. These membrane-destabilizing peptides usually derive from viral proteins responsible for the endosomal escape of these pathogens [295]. Other approaches have considered the incorporation of neutral surfactants that respond by ionizing into positive and surface-active conjugate acids upon luminal acidification. These species can electrostatically destabilize endosomal membranes and exhibit reduced cytotoxicity compared with cationic lipids [296]. Acid-labile lipids have also been used to alleviate the detrimental effects of PEG incorporation on escape efficiency [297]. For instance, Kanamala and colleagues developed PEGylated anionic liposomes that contained acid-labile PEG-lipids, which cleaved under acidic conditions. In consequence, liposomes are shielded by PEG prior to reaching endosomes without significantly impacting their ability to induce the electrostatic interactions responsible for membrane destabilization and fusion [298]. In particular, they reported that the pH-triggered degradation of the PEG layer caused a 1.4-fold increase in endosomal escape when compared to that of liposomes modified with non-cleavable PEG.

The physical changes of pH-responsive coatings and liposomes are also exploited by several drug or nucleic acid delivery systems to induce cargo release from nanocarriers as well as membrane destabilization. Cargo complexation through electrostatic interactions is among the most common strategies due to the ease of cargo delivery upon protonation in acidic environments [299]. Guo and colleagues, for example, designed a pH-responsive multilayered core-shell delivery system by coating IONs with a triblock copolymer, which was subsequently loaded with anti-cancer drugs through electrostatic interactions [300]. The outermost layer was a biocompatible copolymer, the intermediate one was a block copolymer with pendant amine groups and hydrophobic moieties for drug complexation, and the innermost shell was a protonable polymer tightly bounds to the iron oxide core. Drug release was triggered by the disruption of electrostatic interactions after protonation of the carboxylate anion present in the drugs, as well as by the swelling of the protonated inner shell during endosomal maturation [300]. Drug loading through acid-labile linkages (e.g., imide or hydrazone bonds) has also been considered attractive mainly because degradation of these bonds upon luminal acidification ensures efficient drug release inside endosomes [301,302,303]. This strategy has been coupled with other membrane destabilizing strategies to achieve higher escape efficiencies for larger molecules before reaching lysosomal compartments [304].

### 3.5. Enhanced Photoinduced Endosomal Escape via Near-Infrared Irradiation

Photoinduced endosomal escape has been recently reported as an attractive avenue for membrane disruption. In this approach, reactive oxygen species (ROS) and singlet oxygen (^1^O_2_) are generated from photosensitizers [305,306] or photothermal transduction agents [305,307,308] to induce membrane destabilization. These endosomal escape strategies have been termed photochemical internalization (PCI) [306] and photothermal therapy (PTT), respectively [309].

#### 3.5.1. Photochemical Internalization

**Figure 8:**
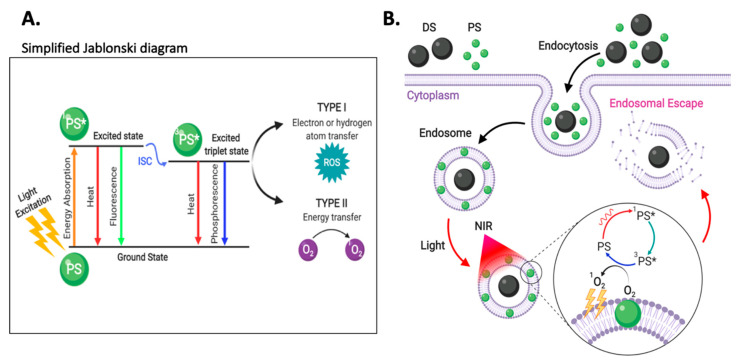
(**A**) Simplified Jablonski diagram showing the different energy transfer events involved in PCI. PS: photosensitizer, ICS: intersystem crossing. (**B**) Schematic illustration of the PCI process. Delivery systems (DS) and photosensitizers (PS) are endocytosed by the cell and colocalized into the endosomal vesicles. PS are mainly localized in the endosomal membranes due to their amphiphilic properties. After NIR irradiation, PS absorb the light energy and transfer it to molecular oxygen, thereby generating highly toxic singlet oxygen. These molecules cause important oxidative damage in the endocytic membranes, which ultimately leads to endosomal escape by their disruption. (Created with BioRender.com).

PCI was developed at the Norwegian Radium Hospital to improve the endosomal escape of various therapeutic nanocarriers sequestered in endocytic vesicles [310]. In this technology, a photosensitizer (PS) is colocalized with the delivery system and subsequently subjected to an external stimulus. As a result, the PS generates chemical or physical alterations by absorbing light and then releasing energy [306], which eventually leads to endosomal membrane disruption [311,312]. Upon light activation, PSs are converted to a short-lived excited state (^1^ P *) that can release the absorbed energy as heat or fluorescence or undergo intersystem crossing (ISC) to a long-lived excited triplet state (^3^ P *) [313]. The triplet state energy can be released as heat or light (phosphorescence) or can be transferred to a target molecule or to molecular oxygen through two different photochemical reactions (Figure 8A) [306,313]. In the type I photoreaction, an electron or a hydrogen atom transfer occurs between the PS and the target molecule, thereby generating ROS [306,313]. Similarly, the type II photoreaction occurs when the energy of the PS is transferred to molecular oxygen to form singlet oxygen (^1^ O_2_) [306,313,314]. The high toxicity of singlet oxygen causes significant oxidative damage in endocytic membranes, which can be mainly attributed to amino acid oxidation, unsaturated fatty acids peroxidation, and cholesterol destabilization [306]. This damage promotes endocytic vesicle disruption, which ultimately results in endosomal escape (Figure 8B). Several researchers have reported that the success of PCI depends on the capability of PSs to enter cells through pinocytic pathways and subsequently localize in endocytic membranes [305,310,312,315]. The most efficient PSs for PCI have an amphiphilic structure, which allows them to intercalate within endocytic membranes [306,312,314]. Some of these compounds include, disulfonated meso-tetraphenylporphine (TPPS2a), disulfonated aluminium phthalocyanine (AlPcS2a), dendrimer phthalocyanine (DPc), 5,10,15-tri(4-acetamidophenyl)-20-mono(4-carboxyl-phenyl) porphyrin (TAMCPP) and tetra(4-sulfonatophenyl) porphine (TPPS4) [256,311,316].

The light activation of PSs is commonly achieved via irradiation with light of a particular wavelength, usually in the visible range [317]. However, UV light activation has also been implemented for uncaging or cleaving nucleic acids or drugs for the delivery of small molecules and gene therapies [317]. However, the use of visible and UV light needed for PCI poses an important limitation due to their low penetration depth through biological tissues [312,317]. Likewise, UV light is widely reported as a phototoxic agent that can be potentially carcinogenic [317]. In contrast, near-infrared (NIR) light exhibits higher tissue penetration compared to UV or visible light [313] but much lower absorption rates in biological tissues [313,318]. As a result, this technology offers reduced phototoxic effect, which makes it a promising alternative for the implementation of PCI at clinical and experimental levels [317,318,319]. In fact, a recent report has demonstrated the biomedical application of NIR PCI through either direct irradiation or via local conversion with the aid of nanoparticles [320].

##### Direct PS activation via NIR Irradiation

In this strategy, PS activation is directly performed by a NIR irradiation source, thus, intermediary molecules or additives are not required [320]. This technology has demonstrated efficient endosomal escape of multiple nano-release platforms in several biomedical applications such as drug delivery, cancer therapy, gene therapy, and imaging [320,321]. Recent reports have explored the combination of IONs with different PSs to enhance the performance of magnetically-guided and photoinduced delivery systems. Bhana and colleagues, for instance, developed a hybrid PCI and PTT technology for cancer therapy using Au-ION nanopopcorns coated with the PS silicon 2,3-naphthalocyanine dihydroxide and stabilized with PEG linked with 11-mercaptoundecanoic acid [322]. The hybrid nanocarrier demonstrated highly efficient photothermal conversion (61%), and a superior PS release rate, as well as complete eradication of tumors without significant systemic toxicity in vitro [322]. Similarly, Hou and colleagues synthesized theranostic nanoparticles by immobilizing IR820 (PS) onto the surface of chitosan-coated IONs for cancer therapy, diagnosis and MRI [323]. The nanoconjugates exhibited significant cellular uptake, and successful endosomal escape, which allowed the annihilation of cancer cells after irradiation with NIR light (808 nm). Additional surface modifications of IONs have demonstrated excellent results for cancer therapy and imaging. In this regard, it is possible to highlight the immobilization of various PS molecules including IR806 on bare IONs [324], multifunctional silica-based IONs coated with (2,7,12,18-tetramethyl-3,8-di(1-propoxyethyl)-12,17-bis-(3-hydroxypropyl) porphyrin (PHPP) [325], AU core-shell IONs coated with reduced graphene oxide for doxorubicin delivery [326], and silica core-shell IONs functionalized with chlorinepyropheophorbide-a (PPA) [327].

##### Upconverted Nanoparticles

This strategy is based on the use of certain chemical substances that can transform low energy photons into high energy ones [320]. This phenomenon can be utilized to convert NIR light into UV or visible light radiation. The emitting radiation, namely, UV or visible, can be used to photo-activate PSs or for uncaging/cleaving nucleic acids or drugs [320,328]. The UV/Vis systems resulting from the conversion of NIR are commonly described as upconverted systems. When their implementation is aided by nanoparticle hosts, they form platforms known as upconverted nanoparticles (UCNPs) [308].

**Figure 9:**
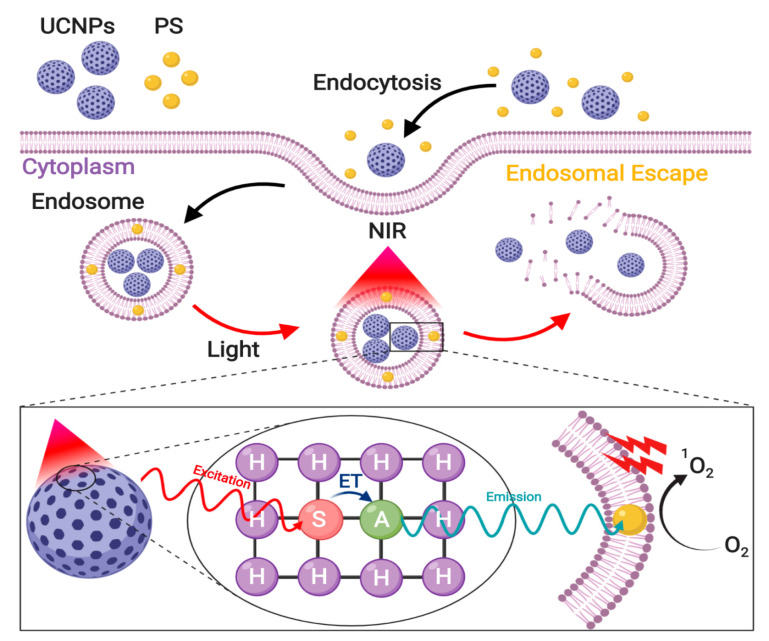
Schematic illustration of the PCI process by using upconverted nanoparticles (UCNPs). UCNPs and photosensitizers (PS) are endocytosed by the cell and colocalized with endosomal vesicles. PS intercalate within endosomal membranes due to their amphiphilic properties. After NIR irradiation, sensitizer ions (S) absorb the energy and transfer it to activator ions (A) capable of emitting radiation (UV or Vis). PS then absorb the energy and transfer it to molecular oxygen, thereby generating highly toxic singlet oxygen. These molecules cause important oxidative damage in the endocytic membranes, which ultimately leads to endosomal escape by their disruption. ET: energy transfer, H: host matrix (Created with BioRender.com)

UCNPs are usually composed of at least three fundamental components, namely, sensitizer ions, activator ions, and a physicochemically stable host matrix [328]. Sensitizer ions absorb the energy from the NIR irradiation and transfer it to the activator ions, which can ultimately emit their characteristic luminescence (Figure 9). Several ions can be used as sensitizers in the UC systems; however, Yb^3+^ has been the most widely implemented [329]. The selection of activator ions mainly depends on the emission wavelength of interest. For instance, activators such as Tm^3+^, Er^3+^, and Ho^3+^ can be used to produce UV/blue, green and red radiations, respectively [329]. The most versatile activators are Tm^3+^ ions as they can produce emissions in the UV (∼350 nm), NIR (∼800 nm), and visible wavelength ranges [328]. The host matrix selection mainly depends on its physicochemical stability and the presence of a crystalline structure capable of holding the added dopant ions [328].

Hybrid ION/UCNP systems have been widely applied in cancer therapy, MRI and diagnosis, gene therapy, and drug delivery [308]. In general, the most prevalent host matrices for iron oxide core-shell nanoparticles include Y_2_O_3_, Y_2_O_2_S, LaF_3_, BaYF_5_, NaYF_4_, and NaGdF_4_, which have been doped with Yb^3+^/Tm^3+^ and Yb^3+^/Er^3+^ ions [230,231,232,233,234,235,236,237,238,239,240,241,242,243,244,245,246,247,248,249,250,251,252,253,254,255,256,257,258,259,260,261,262,263,264,265,266,267,268,269,270,271,272,273,274,275,276,277,278,279,280,281,282,283,284,285,286,287,288,289,290,291,292,293,294,295,296,297,298,299,300,301,302,303,304,305,306,307,308,309,310,311,312,313,314,315,316,317,318,319,320,321,322,323,324,325,326,327,328,329,330,331,332]. For instance, the early diagnosis of Alzheimer’s disease has been attempted with the aid of ION/UCNPs (BaYF_5_ host matrix with Yb^3+^, Er^3+^ ions) conjugated with the Aβ_0_ aptamer (DNA1) and the complementary oligonucleotide of the Aβ_0_ aptamer (DNA2) [333]. ION/UCNPs (NaGdF_4_ host matrix with Yb^3+^, Er^3+^ and Tm^3+^ ions) based on hollow carbon spheres have also been employed for tumor elimination and MRI [334]. Similarly, ION/UCNPs with a Mn^2+^ doped NaYF_4_:Yb/Er outer shell showed promising potential for MRI diagnostics [335]. Importantly, the ION/UCNPs usually include biocompatible outer shells that improve colloidal stability and can be also guided with the aid of magnetic fields [308].

#### 3.5.2. Photothermal Therapy

**Figure 10:**
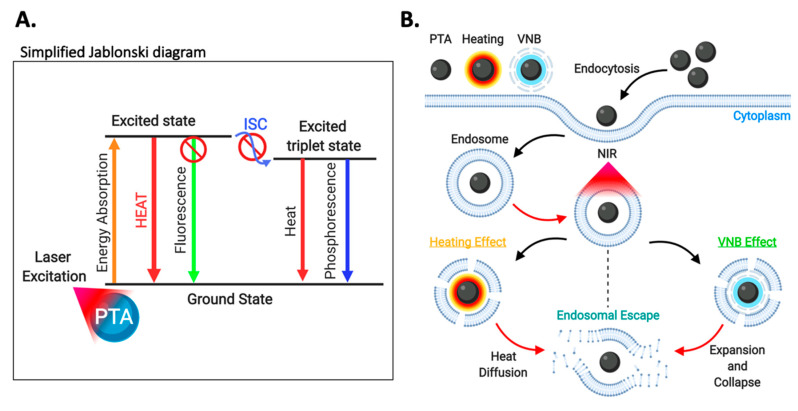
(**A**) Simplified Jablonski diagram describing the different energy transfer mechanisms involved in PTT. PTA: photothermal transduction agent, ICS: intersystem crossing. (**B**) Schematic illustration of the PTT process. Photothermal transduction agents (PTAs) are taken up by endocytosis and trapped into the endosomes. After NIR irradiation, PTAs absorb the light energy and transform it into heat, which could lead to endosomal escape by two major mechanisms. In the first one, also known as the heating effect leads to the destabilization of endosomal membranes by a localized increase in temperature. In the second one, the released heat is high enough to generate a vapor layer surrounding the PTAs such that it expands as a vapor nanobubble (VNB) that eventually collapses to induce endocytic membrane disruption. (Created with BioRender.com).

Photothermal therapy (PPT) is an alternative technique that relies on a photothermal transduction agent (PTA) to convert light into heat to increase the temperature in a localized manner (Figure 10A). This might lead to endocytic vesicle disruption and, consequently, to increased endosomal escape [309,320]. This approach makes use of a non-radiative relaxation pathway of photoexcitable molecules called internal conversion, where the light energy is transformed and dissipated as mechanical and thermal energy [320]. The photothermal effect can lead to endosomal escape mainly by two mechanisms (Figure 10B). The first one, known as the heating effect, is a light-activation where a PTA is excited to a high energy level that releases heat to destabilize endo-lysosomal membranes [336]. The second one occurs at very high heat releasing levels capable of generating a vapor layer surrounding the PTA. This vapor expands like a bubble and eventually collapses, thereby inducing endocytic membrane disruption. In this mechanism, the endosomal escape is attributed to mechanical energy dissipation (expansion and collapse of the vapor nanobubbles) and not to the heat diffusion. This has been demonstrated by the negligible heating of the intracellular environment [308,336].

PTAs are typically classified into organic and inorganic compounds. Inorganic materials include noble metals (e.g., Au, Ag, Pt, and Pd) [337], metal chalcogenides [309], carbon-based nanomaterials (e.g., graphene and carbon nanotubes) [307,309] and other two-dimensional structures (e.g., black phosphorus, nanosheets, boron nitride, and MXenes) [309,338]. In contrast, organic PTAs include small molecules such as porphyrin and cyanine [309] as well as semiconducting polymer NPs (SPNPs) [309,339]. Although inorganic PTAs usually exhibit superior photothermal stability than organic PTAs, they have demonstrated limited biodegradability and biocompatibility. As a result, the appropriate selection of a PTA for a particular PPT application is still challenging and the focus of intensive research [309].

The ample variety of PTAs and their unique physicochemical properties are useful for a number of applications in the biomedical field including biological imaging, drug delivery, cancer therapy, and hyperthermia [308]. Recent work by Farani and colleagues showed a promising delivery system based on the PEGylation of graphene-coated IONs for the delivery of doxorubicin for cancer therapy. They reported excellent cellular uptake, high biocompatibility, and remarkable drug release efficiency. In addition, they highlighted the use of IONs for the development of efficient photothermal systems that can be guided by external magnetic fields [340]. Similarly, Lu and colleagues synthesized ION-Au core-shell nanoparticles as delivery vehicles of Cetuximab (C225) for the treatment of human glioma (U251) cells. They reported that upon PTT application, apoptosis was triggered for U251 cells in vitro, and tumor growth was fully suppressed in vivo [341]. Moreover, Seabra and colleagues put forward a number of iron oxide-based nanocarriers for cancer therapy with remarkable performance including Au-coated IONs and IONs coated with both Au and reduced graphene oxide [308].

## 4. An Overview of ION-Mediated Transfection in Gene Editing

Gene therapy refers to the correction of damaged or missing genes in an organ or tissue by the introduction of exogenous DNA sequences to the defective cells [342]. The modification of cellular DNA carried out in gene therapy is known as cellular transfection [343]. The delivered genetic material needs to pass several cell barriers in order to reach the nucleus. These barriers include the PM, the endosomal membrane (if the nanoparticles enter the cells by endocytosis), and the nuclear envelope [342,344]. Inefficiency to cross the nuclear envelope is one of the principal reasons for low transfection efficiencies [342]. In this regard, it has been shown that only 0.001–0.1% of the available nucleotide molecules in the cytoplasm can carry out transfection processes [345]. Moreover, research has shown that once inside the nucleus, the transfection efficiency is a function of the number of therapeutic molecules present in the nuclear space instead of their topologies [346]. Consequently, to ensure high transfection efficiencies it is of paramount importance to have a large input of the delivered molecules within the nuclear region [343].

Recent studies have shown that the success of the insertion of macromolecules in the nucleus largely depends on the stage of cell division [342,343]. In proliferating cells during the G2/M phase period, the transfection process is accomplished more easily because the delivered sequences are able to reach the perinuclear area due to the rupture of the nuclear envelope [343]. In quiescent or non-dividing cells, the main translocation route between the cytosol and the nucleus is through the nuclear pore complex (NPC) [343,347]. The NPC is a dynamic structure [342] composed of nucleoporins and associated nuclear and cytoplasmic filaments [343,348]. Transport through NPC involves passive and active mechanisms that are initiated according to the molecular weight or size of translocating molecules [342,343]. Passive transport is used by ions and small proteins (<40 kDa) with a diameter smaller than 9 nm [342,346], whereas larger molecules with a maximum diameter of 39 nm (<60 MDa) internalize through active transport [345]. Since most of the transfection molecules, including plasmids and drug compounds, have a size between 2–10 MDa, gene therapy in non-mitotic cells depends on nuclear active transport [345,348].

**Figure 11:**
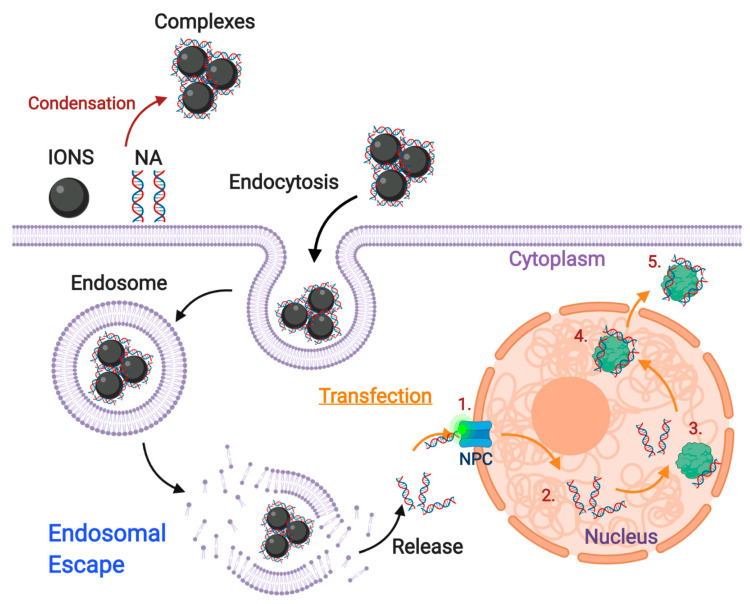
Schematic illustration of the transfection mechanism. After the endosomal escape of the vehicles, the cargo (usually DNA) is released into the cytoplasm. The cargo interacts with the nuclear pore complex (NPC), where importin proteins activate to mediate nuclear internalization (**1**). Inside the nucleus, internalized molecules interact with nuclear structures (**2**). Subsequently, the remaining molecules bind to RanG proteins (**3**) for their recycling (**4**) and release into the cytoplasm (**5**). (Created with BioRender.com).

Active nucleocytoplasmic transport is mediated by importin proteins, which are nuclear receptors of the karyopherin β family. This protein family is in charge of the nuclear internalization of ribosomal and mRNA binding proteins [342]. Activation of importin proteins requires interaction between them and nuclear localization signal (NLS) sequences [348]. However, internalized molecules can bind to RanG proteins within the nuclear space, thereby leading to their dynamic recycling and release back to the cytosol (Figure 11) [345,348]. This emphasizes the importance of achieving a high nuclear concentration of therapeutic molecules for an effective transfection. The amino acid sequences found in NLS exhibit a strong positive charge [348], which has motivated the search for new sequences with such character [345]. This section is therefore dedicated to discussing modification of IONs to allow their utilization as vehicles for gene therapy.

### 4.1. Cationic Peptides and Polymers

Cationic polymers are widely used for the design of nanoparticle-based gene therapies. Their positive surface charge is useful to promote interactions with negatively charged DNA and RNA molecules. The formed complexes are known as polyplexes and are maintained via electrostatic interactions [349]. Polyplexes provide excellent protection for nucleotide sequences from nucleases and allow cellular uptake via pinocytic pathways and posterior endosomal escape via the proton-sponge effect [350]. Once inside the cytoplasm, the DNA/RNA is released from the polymer, albeit at relatively low rates due to the exceedingly high strength of such an interaction [350]. To address this issue, polymers have been modified with hydrophobic moieties, which have been proven effective to lead to higher volumes of genetic material available for nuclear entry [350].

Among cationic polymers, perhaps PEI shows the highest protective effects over plasmid DNA (pDNA). In this regard, Rohiwal and colleagues demonstrated that after exposure to 10% (v/v) fetal bovine serum, naked pDNA degraded within 24 h while the polyplex PEI-pDNA remained stable beyond the same timespan [251]. For this reason, IONs have been modified with PEI to improve transfection efficiency, as demonstrated by Kami and colleagues, who achieved an 8-fold increase for episomal vectors after exposure to a magnetic field [351]. Arsianti and colleagues systematically investigated the effect of ION-PEI-DNA complexes in transfection efficiency by varying the component arrangement on the nanoparticles. The highest cellular uptake and gene expression was observed for the ION+PEI/DNA (where PEI/DNA complexes adhere to bare IONs) which was attributed to enhanced gravitational and magnetic aided sedimentation onto the adherent cells [352].

PEI has also been used in combination with NLS peptides. For instance, Song and colleagues generated ternary complexes of PEI-coated IONs, pDNA, and the endosomolytic TAT peptide through electrostatic interactions. Upon exposure to a magnetic field, the transfection efficiency both in vitro and in vivo increased considerably. This has been thought to be the result of the high cellular penetration and nuclear localization capacities of the TAT peptide [253,353]. Peptides derived from simian vacuolating virus 40 (SV40) have also been used for transfection applications. Vernon and colleagues modified pDNA with the SV40-derived DNA targeting sequence (DTS), which consisted of binding sites for numerous transcriptional factors such as AP1, AP2, AP3, AP4, NF-κB, Oct-1, and SP1 [354]. IONs modified with pDNA-SV40DTS have shown strong interactions with importins to generate nuclear pore complexes in non-dividing SH-SY5Y cells [354]. This led to significantly enhanced transfection efficiencies, as analyzed by flow cytometry and fluorescent imaging, compared with the vehicles in the absence of the DTS. This was the case for different commercially available ION vectors including PolyMag Neo, nTMag, and Neuromag [354].

NLS peptides have also been used to allow penetration of the whole IONs to the nucleus. Wang and colleagues designed a peptide based on both the large T antigen of SV40 and a receptor-mediated endocytosis signal peptide. This new peptide was able to facilitate the penetration of whole nanoparticles into the nucleus of HepG2 cells [355]. Previous work from our group also demonstrated the nuclear penetration of whole PEA-ION nanobioconjugates modified with the peptide Buforin II (i.e., BUF-II-PEA-IONs). This activity was attributed to the presence of BUFII and the ability of the PEA surface extensor to maintain BUF-II’s cell-penetrating capabilities [131].

### 4.2. Cationic Lipids

Cationic lipids have been extensively used for the delivery of gene therapies mainly due to their amphiphilic character, cost-effectiveness, and high biocompatibility [270,356,357]. The structure of cationic lipids comprises four functional domains, namely, a hydrophilic head-group, a hydrophobic portion, a linker bond, and a backbone. The head-group is positively charged and responsible for interactions with NPC. The hydrophobic domain is made up of steroid or alkyl chains (saturated or unsaturated). The linker and the backbone are spacers between the backbone and the hydrophobic domain and the head-group and the hydrophobic domain, respectively. Additionally, the backbone domain is used as a scaffold for cationic lipid construction [358,359]. According to the backbone composition, cationic lipids can be divided into two categories, namely, glycerol-based, and cholesterol-based [270,358].

Despite the great variety of lipids that are potentially available for the development of gene therapy carriers, none of them completely fulfills ideal vehicle characteristics. This is mainly due to the variability of the transfection pathway, which is largely influenced by changes in the charge density of the polar head groups, the length of hydrophobic tails, and the type and density of linker groups. These structural features might also impact the transfection efficiency, biodegradability, stability, and cytotoxicity of cationic lipids [357,358,360]. As a consequence, a specific relationship between the molecular structure of cationic lipids and their transfection efficiency is still elusive [360]. A strategy to improve the cell-penetrating potency of cationic lipids is to combine them with neutral or zwitterionic lipids or polymers [357].

Cationic lipids based on 3ß-[N-(N′,N′-dimethylaminoethane)-carbamoyl]cholesterol (DC-Chol) or N-(1-(2,3-dioleyloxy)propyl)-N,N,N-trimethylammonium (DOTMA) are the preferred choice for delivery systems [358]. DC-Chol is the most popular cholesterol-based cationic lipid [356] while DOTMA is that of the glycerol-based family [361]. Both molecules have attracted significant attention as envelopes for ION-based vehicles mainly due to their ease of generating complexes with oligonucleotides [361], high rate of transfection, and biocompatibility [356]. Du and colleagues encapsulated iron oxide-oleic acid-DMSA nanoparticles into bilayered liposomes formed by DC-Chol and cholesterol. The formed encapsulates maintained magnetic responsiveness and demonstrated high gene-binding affinity and transfection rates [362]. Similarly, Zheng and colleagues developed DC-Chol based liposomes loaded with IONs in a tartaric acid matrix for gene delivery treatments. The vehicle led to high transfection rates in THLE-3 cells [363]. Hirao and colleagues synthesized cholesterol-based magnetoliposomes for plasmid DNA delivery in human osteosarcoma Saos-2 cells. Their results showed that by adding the lipid envelope, the transfection rate improved by nearly 3.5-fold when compared with uncoated IONs [364]. Preiss and colleagues synthesized monodispersed lipid-coated iron oxide nanoparticles (L-IONs) with a lipidic envelope produced by self-assembled monolayers of cationic DOTMA and anionic polymer PEG. Cationic lipid coatings in equimolar ratios with anionic PEG–lipids showed superior cell viability, cellular uptake, and transfection efficiencies when compared with lipid coatings in the absence of PEG [130].

### 4.3. Dendrimers

Dendrimers are a unique class of synthetic polymers that exhibit well-defined branched tree-like topological structures [365,366]. They are usually composed of single or multiple layers of highly ordered branching units, termed dendrons, radiating from a central core with a high density of terminal groups located on the final dendron layer (dendrimer surface) [365,367]. These polymers are precisely synthesized in a stepwise and controlled manner to obtain materials with the desired size, shape, number of dendrons, surface charge, and type of terminal groups [366,367]. This versatile synthesis along with a great variety of initiator cores, branching units, and multiplicities, allows precise tuning of the physicochemical properties of dendrimers. Dendrimers have therefore attracted significant attention for several biomedical applications such as cancer therapy, drug delivery, treatment of inflammatory diseases, MRI imaging, antiviral therapies, and gene delivery [365,366,367,368].

In particular, properties such as their well-defined chemical structures, high density of terminal groups, ease of surface modification and biocompatibility, have drawn much attention to dendrimers for the development of gene and drug delivery systems [365,369,370]. Specifically, the easy conversion of terminal groups into amine groups with a positive charge, makes these polymers an effective platform for nucleic acid condensation through electrostatic interactions [365]. Moreover, dendrimers are effective internalization vehicles that can protect nucleic acids from enzymatic degradation [368]. Furthermore, some dendrimers such as polyamidoamine (PAMAM), poly(propyleneimine) (PPI), and poly(etherimine) (PETIM) present a high density of tertiary amine groups that can lead to endosomal escape via the proton-sponge effect [365].

Although dendrimers have demonstrated excellent potential as nonviral platforms for gene therapy [368,370,371], recent developments have shown enhanced cellular uptake and transfection rates by combining them with nanostructured materials. Examples of such materials include IONs, gold nanoparticles, carbon nanotubes, silica nanoparticles, and polymeric nanocomposites [371,372]. Specifically, the use of dendrimer-based IONs has been broadly studied due to numerous advantages such as increased DNA/RNA binding sites per dendrimer molecule, biocompatibility, DNA compaction ability and enhanced targeted delivery through magnetic guidance [368,372,373,374,375]. Taratula and colleagues developed a PPI dendrimer (G5) coated ION co-immobilized with PEG and LHRH peptide (cancer specific-targeting moiety) for the delivery of multifunctional siRNA for cancer therapy. They reported an efficient suppression of BCL2 (B cell lymphoma) mRNA that, in turn, led to a significant enhancement of the in vivo antitumor activity of the anticancer drug cisplatin [376]. Similarly, Xiao and colleagues synthesized an innovative plasmid DNA delivery system based on the use of PAMAM dendrimers-poly(styrene) sulfonate coated IONs. They found that transfection rates and cellular uptake are highly conditioned by the ratio between the number of primary amines in the PAMAM dendrimer and the number of phosphate groups in the pDNA (N/P). Importantly, dendriplex-coated IONs formed by generation six dendrimers at an N/P ratio of 10 exhibited the highest luciferase protein reporter gene expression and uptake rate in NIH 3T3 cells (murine fibroblast) [377].

A more recent work by Albukhaty and colleagues described a novel nanocarrier based on the immobilization of poly-L-lysine (PLL) dendrimers on IONs (ION-PLL) for the delivery of the pro-brain-derived neurotrophic factor (BDNF) gene into neural stem cells (NSCs) [157]. The obtained BDNF levels were five times higher in the transfected cells compared to the untransfected ones. Furthermore, the nanocarriers were able to maintain a constant supply of the BDNF gene in the NCSs, which is a remarkable result for future application of this technology in the treatment of neurodegenerative diseases such as Parkinson’s, Alzheimer’s, Huntington’s and amyotrophic lateral sclerosis [157]. A similar development by Thomas and colleagues showed micelles loaded with IONs modified with PLL/hyaluronic acid (HA) for pDNA delivery with potential application in MRI and cancer theranostics. They reported remarkable transfection efficiency of the reporter luciferase plasmid and GFP in CT-26 cells (murine colon cancer) and enhanced MRI contrast [378].

### 4.4. Enhancing the Transfection Process with Magnetic Fields

**Figure 12:**
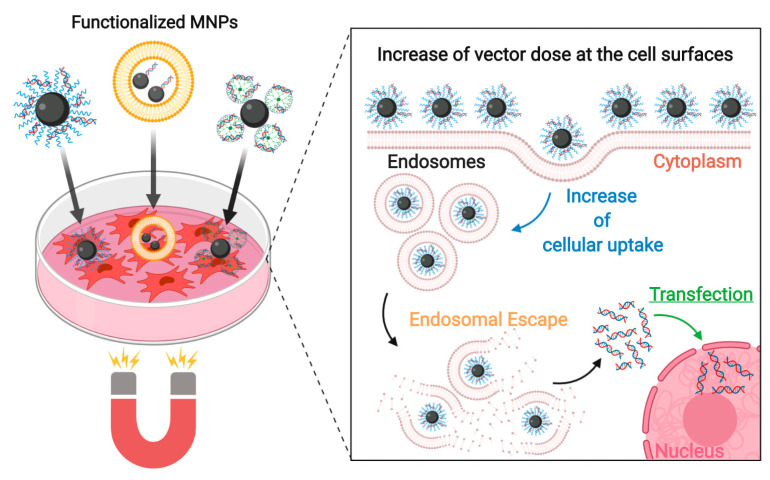
Schematic of the magnetofection principle. Under the effect of a magnetic field, functionalized IONs are guided directly to target cells. This generates an increase in the vector availability at the cell surfaces that leads to an increase in cellular uptake. Endosomal escape occurs by the action of the specific molecules used to functionalize IONs. High transfection rates can be achieved: More nucleic acids loaded IONs into the cytoplasm leads to an increase of free nucleic acids generating more efficient transfection rates. (Created with BioRender.com).

IONs have been widely studied in gene therapy mainly because of the possibility they confer to selectively attach and transport targeted molecules to a specific location under a magnetic field [374]. The use of magnetic fields for enhancing transfection rates was first described at the beginning of the 2000s by Christian Plank’s research group in Munich [379]. This approach, known as magnetofection (MF), is based on the application of magnetic fields to favor the sedimentation of nanocarriers and concentrate them in a targeted location. The success of MF is attributed to an increase of vector dose at the cell surface level, which leads to an increase in the cellular uptake (Figure 12) [380]. As more nanocarriers penetrate into the cells, more DNA/RNA cargoes will be available for the therapy. Nevertheless, it is important to highlight that an enhancement in transfection is not only dependent on the level of cellular uptake but on the number of molecules able to escape endosomes. Planck and colleagues also showed that the cellular uptake dynamics is mainly dependent on the type of modifications introduced on the IONs surface. This was evidenced by the negligible improvement in uptake even when a higher concentration of bare nanocarriers was observed on the cell surfaces as a result of the application of a static magnetic field [380]. Similar results have been observed by other research groups [381,382]. In contrast, other publications have shown that as opposed to static magnetic fields, the oscillatory ones facilitate uptake and led to superior MF [380,383,384,385]. However, the evidence is not compelling enough to elucidate whether this is also the case for endosomal escape. In consequence, the vehicles require functionalization with an effective endocytic disruption agent [386]. In this regard, perhaps the most attractive molecules for facilitating endosomal escape in MF are cationic polymers, particularly PEI [381,386]. Nevertheless, escape has been also observed for molecules such as peptides [387], dendrimers [372], and cationic lipids [388].

Over the past 20 years, the outstanding potential of MF to significantly improve the transfection rates in several cell lines has been demonstrated by a large body of literature. For example, Cui and colleagues developed a gene delivery platform based on PEGylated DNA-PLGA-PEI IONs for difficult-to-transfect neurons (primary hippocampal neurons) [389]. After the application of an external magnetic field, the transfection efficiency increased from 5.8% to 6.5%, and was replicated in vivo. This approach showed a significant improvement as previous reports led to only 5% transfection efficiency [389]. Similarly, Hryhorowicz and colleagues presented the development of a PEI-IONs delivery system for CRISPR/Cas9. They showed that after delivery in porcine fetal fibroblasts, the MF efficiency increased 3.5-fold compared to the conventional lipofection method [390]. Huang and colleagues synthesized a DNA-PEI-HA-ION gene delivery system for cancer therapy in human mesenchymal stem cells (hMSCs) based on expressing the tumor necrosis factor-related apoptosis-inducing ligand (TRAIL). Results showed that the transfection efficiency increased from 20% to 65% under the magnetic field attraction. In vivo experiments in Cg-Foxn1nu/CrlNarl mice confirmed suppression of human glioma (U87MG) by magnetic ternary nanohybrid-transfected TRAIL-expressing hMSCs [391].

Cen and colleagues also showed that the transfection efficiency of PEI-ION/pDNA complexes into human osteosarcoma cells (MG-63) was significantly increased by the application of a uniform magnetic field (about four-fold increase) [392]. Moreover, they found that by changing from a non-uniform to a uniform magnetic field, the efficiency doubled to 42.1% [392]. In the same way, Vaca and colleagues studied the effect of MF time and the culture-to-magnet relative position on the transfection efficiency and cellular uptake [381]. They found that magnetic nanoparticles tend to arrange into concentrated regions with ring-like or circular shapes depending on the magnet location, which, in turn, led to different transfection levels [381].

## 5. Concluding Remarks

Over the past two decades, IONs have attracted significant attention to the development of cell-penetrating vehicles mainly due to the possibility of engineering their surfaces in a relatively easy manner to incorporate different chemistries, which grants them the ability to interact with different physiological barriers and microenvironments. This attractive feature is further potentiated with their strong magnetic responsiveness, which makes them amenable for guided transport to target sites via both static and oscillating magnetic fields that grant precise control over their spatial distribution. Moreover, the application of magnetic fields has demonstrated promising results for increasing the availability of carriers at the cell surface. However, despite the progress made towards making them specific for the needs of particular therapeutic applications, only a few of them have translated into clinical applications [102]. This has been partially attributed to the limited understanding of the ultimate fate and long-term effects of the IONs in vivo. For this reason, numerous research efforts have been dedicated to a deeper understanding of the mechanisms for cell internalization and intracellular trafficking. The ultimate goal is to identify optimal strategies for the rational design of IONs capable of delivering therapeutic cargoes at high rates.

In this regard, some of the most implemented strategies to enhance ION uptake include the incorporation of charged coatings to promote adsorptive electrostatic interactions with cellular membranes and tethering ION surfaces with target-specific ligands that are recognized by membrane receptors in tissues of interest. Moreover, a thorough understanding of the internalization of IONs revealed that the most prevalent route is clathrin-dependent, although caveolae and clathrin- and caveolin-independent mechanisms may also contribute. This has encouraged the engineering of a number of smart nanocarriers that can enter cells within endosomal compartments and escape them by making use of protonable or cationic polymers (e.g., PEI, pDMAEMA, chitosan), peptides (e.g., Buf II, TAT, GALA and INF7), proteins (e.g., OmpA) and liposomes, as well as PSs (e.g., IR820, IR806, PHPP and PPA) and PTAs (e.g., Au and graphene) to destabilize endosomal membranes. Accordingly, optimizing both ION uptake and endosomal escape capabilities requires the strategical design of multifunctional surfaces that adequately interact with different environments at the cellular level. A close inspection of the optimal surface modifications reviewed for each purpose reveals that ION surfaces can be tailored to address both objectives. For instance, IONs could be decorated with a protonable coating (e.g., polymer, lipid) that promotes endosomal escape, and subsequently tethered with a targeting agent that promotes its receptor-mediated endocytosis in a specific tissue. Alternatively, a charged coating could be incorporated to promote ION internalization, and further decoration with a pH-responsive peptide may also favor endosomal rupture upon environmental acidification. The incorporation of cell-penetrating peptides is also a plausible alternative as they have shown exceptional membrane-penetrating abilities, which can be exploited both at the PM and the endosomal membrane, as well as to induce endocytosis upon charged interactions with negatively charged membrane proteins. However, certain combinations arise that may present conflicting outcomes; therefore, it is imperative to adequately select ION architecture to encourage an efficient intracellular delivery and targeted cargo release (i.e., within the organelles of interest). Moreover, the impact of the chosen immobilization method for the cargo (e.g., drugs or gene vectors) on ION surfaces should also considered with special attention to assure an optimal balance between cargo loading and ION interactions with their environment.

However, despite the important advances in the design of potent nanoparticle carriers, efforts should also be focused on the development of more effective technologies for assessing nanoparticle interactions at the cellular level in vivo. This is particularly important since the lack of validating strategies considerably hampers the assessment of their long-term systemic performance, which is crucial for identifying potential features that limit their functionality and for validating their stability and toxicity profiles. Future studies should be focused on obtaining more robust biocompatibility analysis including long-term evaluation of the cellular and systemic impact of the nanovehicles, which is rather difficult to predict from in vitro experiments. This will contribute to a better understanding of the different molecular mechanisms that are involved in the long-term cellular response to such technologies to avoid potential undesirable effects such as imbalanced homeostasis, DNA damage, oxidative stress, or inflammation. Moreover, much work is still needed for the design of devices that dynamically adjust magnetic gradients to precisely concentrate the nanovehicles in locations of interest, especially when the magnetic guidance is intended for in vivo systems. This, in turn, allows the translation of superior nanoparticle systems towards promising theranostic approaches in clinical settings.

IONs hold much promise for the engineering of next-generation cell-penetrating vehicles for highly targeted and smart therapies. Much emphasis on preparing multifunctional interfaces with the capability of facilitating non-invasive routes for the treatment of multifactorial conditions at low dosage regimes. This with the purpose of ultimately increasing the quality of life of patients.

## Figures and Tables

**Table 1:** IONs with cationic and anionic coatings that are internalized through adsorptive interactions with the PM.

Coating	Structure	Zeta Potential (mV)	Hydrodynamic Diameter Water (nm) ^1^	Hydrodynamic Diameter Serum (nm) ^1^	Cell Viability (24 h)	Main Endocytic Mechanism(s)	Internalized Tissue	Ref
**Cationic**								
Chitosan (CS)	Core-shell	~4.2	~122.4	Not reported	≥90% at 30 µg/mL NPs ^a^	Clathrin-dependent	Rat NSCs	[97]
CS-thioglycolic acid	Core-shell	21 ± 5.27	94 ± 20	91 ± 8 nm	≥80% at 300 µg/mL NPs ^a^	Not specified	Human umbilical cord EPCs	[127]
Poly(vinylalcohol/vinylamine)	Core-shell	Positive	~24	Not reported	~100% (up to 123 µg/mL Fe) ^a^	Clathrin-dependent	Me300	[128]
								[129]
diethylaminoethyl-dextran (DEAE-DEX)	Core-shell	~26	~150	Not reported	≥90% (up to 500 µg/mL Fe) ^a^	Clathrin- and caveolin-independent Macropinocytosis	A-549	[130]
PEI-Zonyl FSA/DNA	Core-shell	~52.2 (w/o DNA)	144 ± 0.2	Not reported	≥80% (up to 0.1 µM Fe)^b^	Caveolin-dependent Clathrin-dependent	HEK293	[131]
PEI-Pluronic F-127/DNA	Core-shell	~61.7 (w/o DNA)	160 ± 1.4	Not reported	≥80% (up to 0.05 µM Fe) ^b^	Caveolin-dependent Clathrin-dependent	HEK293	[132]
Lactosylated N-alkyl-PEI2k	Micellar	~28.7	75 ± 6	Not reported	~100% (up to 15 µg/mL Fe) ^b^	Not specified	RAW 264.7	[122]
PEI-stearic acid/PEG-poly(L-glutamic acid)	Polymeric nanosphere	~8	150 ± 25	Not reported	~100% (up to 6.3 µg/mL Fe) ^b^	Not specified	MSCs	[126]
PEI/siRNA	Core-shell	~25.7 (w/o siRNA)	~43.56	Not reported	≥90% at 2 µg/mL NPs (w/o siRNA) ^a^	Not specified	U-87 & U-251	[98]
					≤50% at 2 µg/mL (anti-tumor siRNA) ^a^			
PEI-decorated poly(glycidyl methacrylate)	Polymeric nanosphere	Positive	~160	Not reported	~100% (up to 250 µg/mL NPs) ^b^	Clathrin- and caveolin-independent	Rat PC12	[132]
PEG-g-PEI/siRNA	Core-shell	34.38 ± 1.66	93.8 ± 0.6	Not reported	Non-significant cytotoxicity	Not specified	SGC-7901	[124]
		15.1 ± 0.64 (siRNA)						
PEI-dextran/miRNA	Core-shell	32.5 ± 0.62 (w/o miRNA)	148.67 ± 1.52	Not reported	≥80% (up to 150 µg/mL NPs) ^a^	Not specified	U2	[133]
PEG-g-Chitosan/PEI/siRNA	Core-shell	19.6 ± 5.7 (siRNA)	111.9 ± 52.4	~115 nm	Non-significant cytotoxicity (concentration not specified)	Not specified	Rat C6	[134]
Lipofectamine-Endoderm	Core-shell	−2.45 ± 0.53 *	~181 (PBS)	Not reported	≥80% (up to 50 µg/mL Fe) ^a,b^	Clathrin-dependent Macropinocytosis	HeLa	[135]
Poly-L-lysine (PLL)	Core-shell	~16.9	~24	Not reported	≥90% (up to 25 µg/mL NPs) ^a^	Not specified	NSCs	[136]
PLL-dextran	Core-shell	50 ± 2	115 ± 30	Not reported	≥80% (at 24 µg/mL NPs) ^a^	Not specified	HepG2	[137]
Maltodextrin		25 ± 1.5	60 ± 13.1	Not reported	Not reported	Clathrin-dependent	16HBE14o	[138]
D6DOM/pDNA	Core-shell	9 ± 1.2	71 ± 12	146 ± 29 nm	≥90% ^a^ and ≥85% ^b^ (up to 47 µg/mL NPs)	Not specified	MKN-74 & NUGC-4	[139]
**gH625**-cysteine-PEG-Cy5.5	Core-shell	~4.08	97.8 ±1.2	Not reported	Non-significant cytotoxicity	Not specified	MDA-MB-231	[140]
**PF14**-SCO/Chitosan	Core-shell	~37	~370	Not reported	Non-significant cytotoxicity	Not specified	HeLa	[141]
**PF1221**-SCO/Chitosan	Core-shell	~23	~420	Not reported	Non-significant cytotoxicity	Not specified	HeLa	[141]
Poly(maleic anhydride-alt-1-decene)-dimethylamino propylamine- **Protamine**/siRNA	Core-shell	30.5 ± 2	~30	Not reported	≥90% (up to 65 nM NPs) ^a^	Not specified	MCF-7, U251	[142]
		26.4 ± 3 (siRNA)						
**Anionic**								
PEG-b-poly(e-caprolactone)-g-poly(acrylic acid)	Core-shell	−29 ± 1.9	208.5 ± 4.6	Not reported	~100% (up to 500 µg/mL NPs) ^a^	Clathrin-dependent	CRL-5802	[143]
DNA-PEG	Core-shell	−25.2 ± 0.8	55.8 ± 7.7	74.7 ± 4.4	≥80% (up to 100 µg/mL NPs) ^a^	Clathrin- and caveolin-independent Phagocytosis Clathrin-dependent Macropinocytosis SR-A involved	RAW 264.7	[144]
Carboxy-dextran	Core-shell	~−8.02	~60.32	Not reported	≥90% (up to 100 µg/mL Fe) ^a^	Clathrin-dependent Macropinocytosis SR-A involved	Human macrophages	[145]
Carboxymethyl- dextran	Core-shell	~−48	45 ± 7	Not reported	Not reported	Macropinocytosis Caveolin-dependent Clathrin-dependent	Caco2	[146]
Dextran sulfate	Core-shell	~−45	~60	Not reported	≥90% (up to 5 mM NPs)	Not specified SR-A involved	BV2	[147]
Silica	Core-shell	~−59	~17	~136	Non-significant cytotoxicity (50 µg/mL Fe)	Caveolin-dependent	HeLa	[148]
PEG-silane	Core-shell	~−14	~30	~157	Non-significant cytotoxicity (50 µg/mL Fe)	Caveolin-dependent Clathrin- and caveolin-independent Macropinocytosis	HeLa	[148]
Carboxilic acid-silane	Core-shell	~47	~30	~133	Non-significant cytotoxicity (50 µg/mL Fe)	Caveolin-dependent	HeLa	[148]
Dimercapto-succinate (DMSA)	Core-shell	−49 ± 2 −9 ± 1 (serum)	65 ± 4	128 ± 54	Not reported	Clathrin-dependent Macropinocytosis	Rat microglial cells	[149]
Dimercapto-succinate (DMSA)	Core-shell	−44 ± 14 −14 ± 5 (serum)	50 ± 2	116 ± 13	≥90% up to 2 mM NPs (6 hrs) ^b^	Clathrin-dependent	Cerebellar granule neurons	[150]
Dimercapto-succinate (DMSA)	Core-shell	Not reported	~10	Not reported	~100% (up to 50 µg/mL NPs) ^a^	Clathrin-dependent Caveolin-dependent Macropinocytosis SR-A involved	RAW 264.7	[151]
Glucose	Core-shell	~−40	40–45 (PBS)	Not reported	≥90% (up to 100 µg/mL NPs) ^b^	Caveolae-dependent	Vero cells	[152]
N-(trimethoxysilyl propyl) ethylenediamine triacetate	Core-shell	−39 ± 3	~8	Not reported	Not reported	Caveolae-dependent	Mouse BMECs	[153]
None	-	~−35	20–200	Not reported	≥90% (up to 50 µg/mL NPs) ^a^	Clathrin-dependent	Caco2	[154]
MamC-DOXO	Core-shell	9.6 ± 1	36 ± 12	11–300 nm	≥90% up to 30 µg/mL NPs (w/o DOXO) ^a^	Not specified	HUVECs, KBV1, HeLa	[155]
		−7 ± 0.3 (serum)			≤50% for more than 10 µg/mL NPs (DOXO) ^a^			
Rhodium citrate	Core-shell	−35 ± 6	120 ± 1	Not reported	Not reported	Clathrin-dependent	MDA-MD231, MCF7	[156]
Citrate	Core-shell	Negative (not specified)	8.7–11	Not reported	Not reported	Clathrin-dependent Caveolin-dependent	HUVECs	[157]

^1^ Hydrodynamic diameter measured with DLS. * Authors explain slight negative surface potential due to uneven binding of lipofectamine and only local positive charges. ^a^ Viability was reported according to metabolic activity of cells and ^b^ membrane permeabilization. D6DOM: DC-6-14 (O,O’-ditetradecanoyl-N-(α-trimethlammonioacetyl) diethanolamine chloride) and 1,2-Dioleoyl-sn-glycero-3-phosphoethanolamine (DOPE) (1:0.4); SCO: Splice correction oligonucleotide; Caco-2: human epithelial colorectal adenocarcinoma; HUVEC: Human umbilical vein endothelial cells; KBV1: multi-drug resistant human cervical cancer; HeLa: Human cervical adenocarcinoma; MDA-MB-231: Breast carcinoma; MCF7: Breast carcinoma; BMECs: Brain microvessel endothelial cells; CRL-5802: Human non-small cell lung cancer; RAW 264.7: Macrophages; BV2: Microglia; U251: Human glioblastoma astrocytoma; MKN-74: Gastric adenocarcinoma; NUGC-4: Gastric adenocarcinoma; MSCs: Mesenchymal stem cells; U87: Glioblastoma cells; PC12: pheochromocytoma neural progenitor; HEK293: Human embryonic kidney; A549: Human lung adenocarcinoma; Me300: Human melanoma cells; U2: osteosarcoma; HepG2: Liver hepatocellular carcinoma; NSCs: neural stem cells; SGC-7901: gastric carcinoma; C6: glioblastoma; EPCs: endothelial progenitor cells.

**Table 2:** IONs decorated with targeting agents for their internalization through receptor-mediated endocytosis in their target tissue.

Target	Main Endocytic Mechanism(s)	Targeting Agent	Coating	Target Cells	Application	Ref
LOX-1 receptor	Clathrin- and caveolin-independent [212]	LOX-1 antibody	Poly(ethylenglycol) (PEG)	Activated foam macrophages	Imaging probe for detecting early diabetic nephropathy (DN)	[213]
		OxLDL	anti-OxLDL-PEG	Activated foam macrophages	Imaging of atheroschlerotic plaque lesions	[214]
Transferrin receptor (TFR)	Clathrin-dependent	Transferrin	Dimercaptosuccinic acid (DMSA)	C6	Imaging probe for glioma	[184]
			Ammoniated glucose-oligosaccharides-FITC	4T1	Not specified	[185,186]
			Chitosan/Doxorubicin (DOX)	U251	Drug delivery	[187]
			Dextran-spermine	BBB (in vivo)	Drug delivery in vivo	[215]
			Poly-L-lisine	HeLa	Not specified	[216]
		RI7217 monoclonal antibody	DSPE-PEG-Muscone/Cholesterol/EPC liposomes	BBB and U87-MG in vivo (Mice)	Drug delivery in vivo	[217]
		OX26 monoclonal antibody	Soy PC/DDAB/mPEG2000-PE liposomes	Rat BCECs in vitro and rat BBB in vivo	Targeted delivery to the brain	[218]
EGF receptor	Clathrin-dependent, Caveolin-dependent, Clathrin-and caveolin-independent	EGF	Amino-dextran	C6	Cancer imaging probe	[188]
			Carboxymethyldextran (CMD)	Caco-2	Not specified	[219]
		Nibotuzumab	Silica	A431	Not specified	[191]
		Cetuximab	PEG-dextran	A431	Imaging probe	[190]
		Short-chain EGFR antibody fragments (ScFv)	Poly(ethylene oxide)-poly(γ-methacryloxypropyl trimethoxysilane)	SK-BR-3 & MDA-MB-231	Imaging probe	[192]
VEGF receptor	Clathrin-dependent, Caveolin-dependent	Bevacizumab	PEO-b-PγMPS-NIR830	4T1	Imaging probe	[193]
		Anti-VEGF	Poly(aspartate)-g-poly(ethylene glycol)-dodecylamine-hydrazone-(adriamycin-levulinic acid) micelles	HepG2	Imaging probe	[194]
Human epidermal receptor 2 (HER-2)	Clathrin-dependent	Trastuzumab	PEG-SH	SK-BR-3	Drug delivery	[195]
		Anti-HER2 affibody	Polybutylacrylate-polyethylacrylate-polymethacrylic acid-NIR830	SKOV3	Imaging probe	[196]
Folate receptor	Clathrin- and caveolin-independent	Folate	PEG	U87-MG	Chemotherapy and hyperthermia	[200]
			No additional coating	22Rv.1, LnCaP	Imaging probe and hyperthermia treatments	[220]
			Polyethilenimine (PEI)	KB	Imaging probe	[201]
			PEG-poly(e-caprolactone)	BEL-7402	Tumor imaging	[221]
LRP1	Clathrin-dependent	Lactoferrin	Poly(maleic anhydride-alt-1-octadecene) (PMAO)	C6	Imaging of brain glioma	[222]
		Angiopep-2	Pluronic-poly(acyrlic acid) (PF12-PAA)	BMECs	Delivery to the brain	[223]
CD44	Clathrin- and caveolin-independent Clathrin-dependent [199]	Hyaluronic acid	Hyaluronic acid-C16	MDA-MB-231, NIH/3T3	Cancer imaging and therapy	[202]
		Anti-CD44	DMSA	Panc-1, MBA-MB-231	Cancer therapy	[205]
			CMD	HNSCC	Cancer hyperthermia	[203]
IGF1 receptor	Clathrin-dependent, Caveolin-dependent	IGF1	Amphiphilic polymer	MIAPaCa-2	Drug delivery in vivo	[224]
		Anti-insulin-like-growth-factor binding protein 7 (anti-IGFBP7)	Dextran-Cy5.5	BBB and U87 MG in vivo	Imaging probe	[225]
uMUC-1	Clathrin-dependent	EPPT1	Streptavidin-conjugated dextran	6606PDA (Mouse)	Cancer theranostic platform	[207]
Membrane-bound matrix metalloproteinase (MMP-2)	Clathrin-dependent, Caveolin-dependent [226]	Chlorotoxin	PEG-g-chitosan/PEI	C6	Imaging probe and siRNA delivery to cancer cells	[136]
Carbonic anhydrase IX (CA-IX)	Caveolin-dependent [227]	M75 monoclonal antibody	Poly-L-lysine (PLL)	CA-IX cDNA-transfected C33a cells	Targeting of hypoxic cells (Cancer)	[208]
CD22	Clathrin-dependent [228]	Anti-CD22	Amphiphilic polymer/PEI	preB-ALL	Cancer therapy for preB-ALL cells	[209]
Cholecytoskinin-2 receptor (CCK2R)	Clathrin-dependent	CCK	DY647-PEG	HEK293 stably expressing CCK2R	Cancer therapy	[229]
αvβ3 integrin	Clathrin-dependent, Caveolin-dependent, Clathrin- and caveolin-independent [230]	RGD peptide	PEG	U87 MG	Imaging probe and drug delivery in vivo	[210]

DSPE: 1,2-Distearoyl-sn-glycero-3-phosphorylethanolamine; EPC: 1,2-distearoyl-sn-glycero-3-ethylphosphocholine; PC: L-α-phosphatidylcholine; DDAB: dimethyldioctadecylammonium bromide; mPEG2000-PE: 1,2-dipalmitoyl-sn-glycero-3-phosphoethanolamine-N-[methoxy(polyethylene glycol)-2000; C6: Brain glioma; 4T1: Breast cancer; U251: Human glioblastoma astrocytoma; BBB: blood-brain barrier; HeLa: Human cervical adenocarcinoma; U87-MG: Brain glioblastoma; BCECs: brain capillary endothelial cells; Caco-2: Colorectal adenocarcinoma; A431: Epidermoid carcinoma of vulva; SK-BR-3: Breast carcinoma; MDA-MB-231: Breast carcinoma; HepG2: Human hepatocellular carcinoma; SKOV3: Human ovarian cancer; 22Rv.1: Primary prostate cancer cells; LnCaP: Lymph node metastasis of prostate cancer cells; K8: Cervical carcinoma infected with Human papillomavirus; BEL-7402: Human hepatocellular carcinoma contaminated with human papillomavirus-related endocervical carcinoma; BMECs: Brain microvascular endothelial cells; NIH/3T3: Breast carcinoma; Panc-1: Pancreas/duct epithelioid carcinoma; HNSCC: Head and neck squamous cell carcinoma; MIAPaCa-2: Human pancreatic cancer; 6606PDA: Mouse pancreatic ductal adenocarcinoma; C33a: Human cervical cancer; PreB-ALL: Precursor B-cell acute lymphoblastic leukemia; HEK293: Human embryonic kidney cells.
